# Experimental setup and image processing method for automatic enumeration of bacterial colonies on agar plates

**DOI:** 10.1371/journal.pone.0232869

**Published:** 2020-06-24

**Authors:** Lola Hogekamp, Stefan H. Hogekamp, Mario R. Stahl

**Affiliations:** 1 Institut für Lebensmittel- und Bioverfahrenstechnik, Max Rubner-Institut, Karlsruhe, Germany; 2 PALAS GmbH, Karlsruhe, Germany; Laurentian, CANADA

## Abstract

Automated colony counting methods have long been known in Microbiology. Numerous methods for automated image analysis have been described and a wide range of commercial products exists. Known advantages are saving cost by reducing enumeration time, automatic documentation, reproducibility, and operator independence. Still, even today the realization of all advantages of automated image analysis makes it necessary to either invest in an expensive, high performance commercial system, or to acquire expert knowledge in image processing. This is a considerable obstacle for many laboratories, and the reason why manual colony counting is still done frequently. This article describes an easy to apply automatic colony counting system–including suggestions for sample preparation–that can be put into operation with basic knowledge of image processing and low budget.

## Introduction

Reliable and exact quantitative analysis of microorganisms in liquids is an important part of the daily work in the authors’ laboratory. Plate counting of, e.g., *Escherichia coli* is a recurring task; the strain *E*. *coli* DH5α in particular is used as biological indicator for the efficacy of UV-C treatment of liquids. The results play an important role in the assessment and design of processing steps.

Plate counting is carried out on samples prepared by surface inoculation according to DIN standard [1]. Samples from a dilution series (e.g., 1:10^3^, 1:10^4^, 1:10^5^) are inoculated on broth plates. The original concentration is calculated from the count of colony forming units (CFU) carried out after a given incubation period. Manual CFU counting is still standard. This, for once, cannot be easily repeated since the colonies will continue to grow unless the plates are kept in cool storage, where space is limited. Also, there is usually no photographic documentation but only the CFU number noted down by the counting person.

Exact counting is easily done for high dilution, at the cost of statistical significance [2, 3]. Also, dilution errors take influence. Assessing at lower dilution provides better statistical safety, but the colonies can be so numerous that they become indistinguishable and the count value becomes subjective [2]. Ideally a sample is assessed at a dilution that leads to a colony count per plate in the order of magnitude of 10^2^. According to Breed and Dotterrer [4], satisfactory results can be achieved at up to 400 colonies per plate.

**Fig 1:**
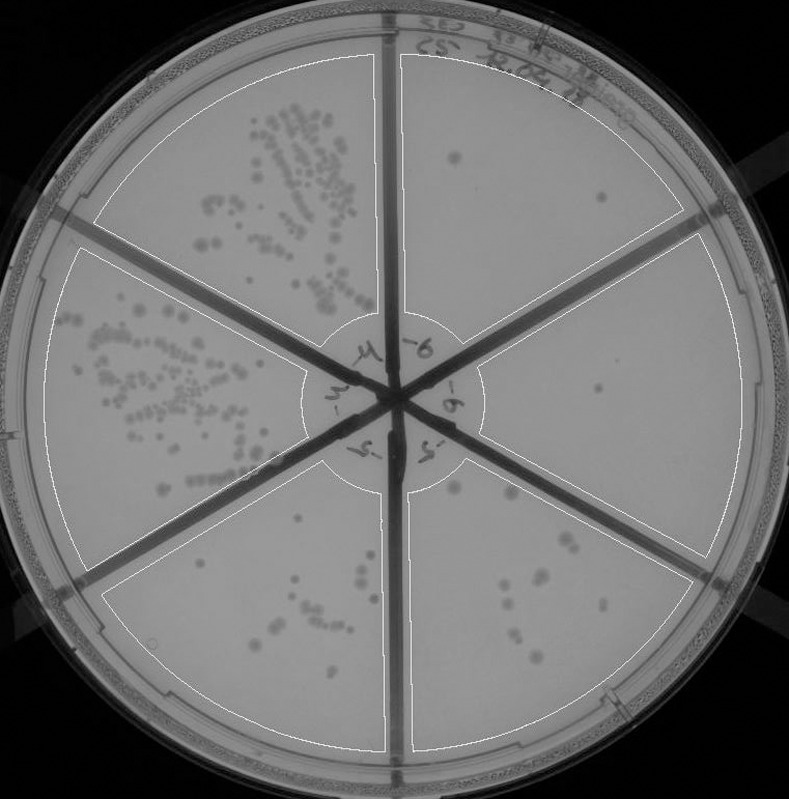
Sample Petri dish. Petri dish split in six segments for inoculation with sample liquids of three different dilution levels (2 repetitions per dilution). Regions of interest for CFU counting outlined.

Manual counting gets even more difficult if, for saving cost, samples from several different dilution levels are inoculated on segments of a single nutrient plate. Fig 1 shows a plate used for enumeration at three different dilution levels. At the lowest dilution the available space is already densely covered by colonies. In such a case it is particularly difficult to determine CFU number without subjective influence and with sufficient statistical reliability. Due to the reduced area per dilution stage colonies must be counted after a short incubation period; counting a large number of small objects, however, is time consuming [5] and tires the assessing person quickly.

**Fig 2:**
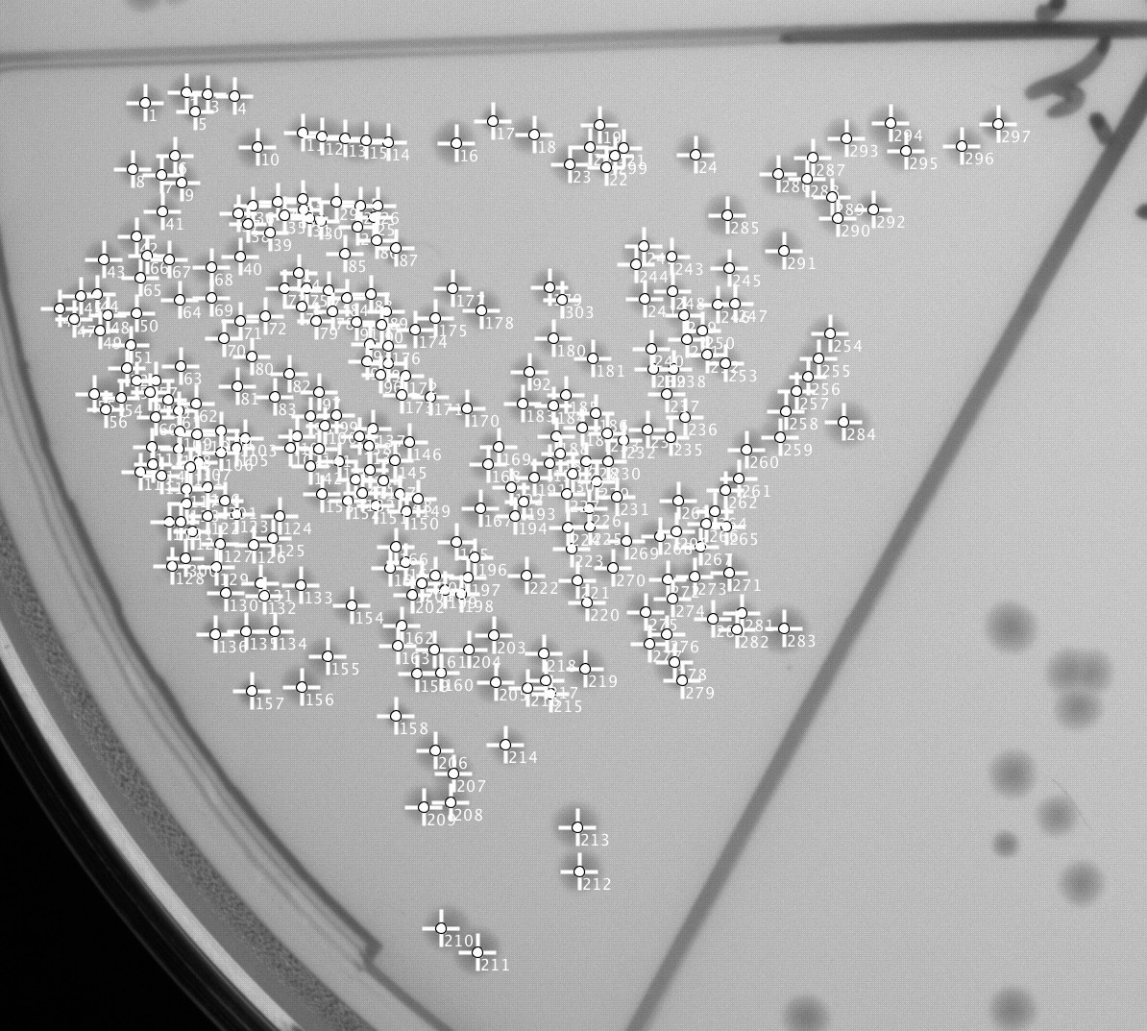
One segment enumerated on a segmented plate. Sample inoculated with *E*. *coli* DH5α, enumerated by point-and-click on screen using ImageJ.

Based on the recommendation of no more than 400 colonies per full plate [4], any segment of a plate split in six segments as shown in Fig 1 should contain no more than 70 colonies. This is, however, difficult to achieve, as the initial microbial load is unknown. Laboratory practice therefore often requires enumeration of non-ideal plates like the one shown in Fig 2 with a count of approximately 300 in the segment.

In principle automated colony counting, as various publications suggest [1, 6–9], increases laboratory productivity considerably. Ideally there will be fewer faults, improved reproducibility, and the results become independent of the training level of the person who carries out the assessment. An image showing the distribution of the colonies is created and stored digitally. If required, this image can be assessed again automatically or manually at any time, which is a significant advantage. Image processing is challenged by variations in colony size, shape, color, contrast, and density as well as by confluence, so some experience in image processing as well as some effort for developing a suitable procedure are required. The effort for finding a solution for the well defined, repetitive task presented here appeared to be, however, manageable.

Initial tests with the public domain image processing software package ImageJ showed that recording a digital image and CFU enumeration in software, executing all steps manually, required less time compared to manual counting for CFU numbers as low as 50 per plate provided that predefined regions of interest (defining the area to be processed) could be used–experienced persons count approximately 2–3 colonies per second by marking & taking notes (see "Results" section below) [8]. At this stage it was estimated that fully automated processing of plate images might lead to a reduction in processing time by one order of magnitude compared to manual counting, with the additional benefit of simultaneous digital documentation. This prospective increase in efficiency was the main motivation for further evaluation of automated counting methods.

At the Max Rubner-Institut automated counting methods had been investigated previously; one major challenge at the time had been satisfactory processing of segmented plates, containing multiple subsamples from different dilution levels. Commercially available colony counters in the midrange price segment, which perform digital imaging and automated counting, had been rated at the time to be unsuitable due to lack of flexibility.

Presently the purchase cost of components for industrial image processing has fallen due to the widespread use of this technology, down to a level which allows the design of high quality systems even with a budget as small as €2,000 –the amount available to us for this project. Software that allows processing of images in seconds even on economically priced personal computers is available commercially as well as in the public domain (on which we focused due to budget restraints).

We thus aimed at developing an image analysis process for counting *E*. *coli* DH5α colonies, which should be simple and robust so that non-expert operators could apply it, preferably without parameter adjustment, even to segmented plates. Part of the project was determining optimized steps for plate preparation that would simplify the subsequent image processing.

## Materials and methods

A review of commercially available products for plate enumeration (optical counting aids, compact automatic counters, and fully programmable image analysis systems) led to the conclusion that commercially available solutions would not meet our budget. While even mid-priced products (order of magnitude 10k €) seemed to be not sufficiently flexible, fully configurable systems appeared to be suitable but far too expensive for our task. We therefore designed a system of our own using individually selected components, as described below.

A review of the features of commercial as well as public domain software for colony counting like NICE (NIST's Integrated Colony Enumerator) [10, 11] and OpenCFU [12] showed that a universally configurable software package would be required. We decided to use ImageJ (version 1.51 64bit for Microsoft Windows^®^), a popular scientific image-processing tool. ImageJ allows step by step manual processing as well as scripting for automation and linking of external modules, and is therefore suitable for experts as well as for beginners who can easily comprehend the effect of a processing step.

### Petri dish preparation

Simplicity of enumeration by image analysis greatly benefits from careful preparation of the Petri dishes. Effects caused by bubbles and foreign particles in the nutrient can, if at all, only be compensated with substantial effort in the processing of the image. One should also aim at constant plate quality, i.e., identical amounts of nutrient and constant layer thickness by precise horizontal alignment when pouring the dishes.

Colony clusters on the rim of the Petri dish are a substantial obstacle for automatic counting (true for manual counting as well). Various, in some cases quite complex algorithms have been published for treating clusters. Since we intended to develop a simple method it appeared to be more worthwhile to try and avoid clusters in the first place by choosing an appropriate inoculation method.

For this purpose lines drawn on the backside of each Petri dish (see Figs 1 and 2) indicate borders, which are not to be crossed during inoculation. If this is duly observed (e.g., with the help of a jig), colonies only grow within the outlined areas. These can subsequently be processed as separate enumeration areas by the image analysis software by defining respective regions of interest (ROIs). The lines facilitate alignment of the dish prior to image recording, making sure that inoculated areas are located within the ROIs of the processing software. In radial direction these regions end a few millimeters short of the rim of the Petri dish, preventing the formation of colony clusters in this area. Avoiding the dish edges also means avoiding the possibly thicker broth layer around the rim of the plate, thus achieving a more uniform background brightness of the image.

### Camera setup for image acquisition

Results of preliminary tests (on digital images recorded in grayscale and RGB color at resolutions between 300 and 800 dpi using a Canon EOS 500D 15 MP SLR camera) suggested grayscale digital imaging of the Petri dishes at a detail resolution of approximately 40 μm in order to analyze colonies down to 0.5 mm diameter. This corresponds to an image of approximately 2,500 by 2,500 pixels for a 100 mm dish. A digital camera with 10 MP sensor (format: 3,840 by 2,748 pixels) of type Basler acA3800-14um with USB3 interface was chosen. This camera is a true grayscale camera, i. e., the sensor is not covered by a RGB filter pattern. Though RGB cameras are frequently used as an image source for grayscale processing, the typically used Bayer filter pattern reduces spatial resolution for the R and B channels to one quarter and halves it for the green channel. Image analysis tasks which do not require differentiation by color are thus preferably solved using grayscale cameras; should filtering by color become necessary again one can still attach a suitable high, low, or band pass lens filter.

Effective use of the resolution offered by a camera’s sensor requires a lens that matches the sensor regarding image circle diameter and contrast performance. The usually applied limit is 50% contrast at a line pairs per mm value corresponding to the sensor's pixel pitch (a pixel pitch of, e.g., 1.6 μm equals 300 lp/mm). The setup shown here employs a Basler C125 lens of 16 mm fixed focal length and f/1.8 maximum aperture. Nominally, contrast performance and image circle of this "5MP" lens are slightly below the optimum for the selected camera. This, however, would mainly affect the corners of the image, which were partially cropped in the first place (the sensor's 3,840 by 2,748 pixels were cropped to 2,748 x 2,748 pixels), and also are not part of the regions of interest since the Petri dish is circular. Furthermore, stopping the lens down to at least f/2.8 improves the contrast performance of the lens and equalizes background brightness as well [13]. This lens-camera combination is therefore well suited for recording circular Petri dishes and, at approximately €500 purchase cost, economically priced.

**Fig 3:**
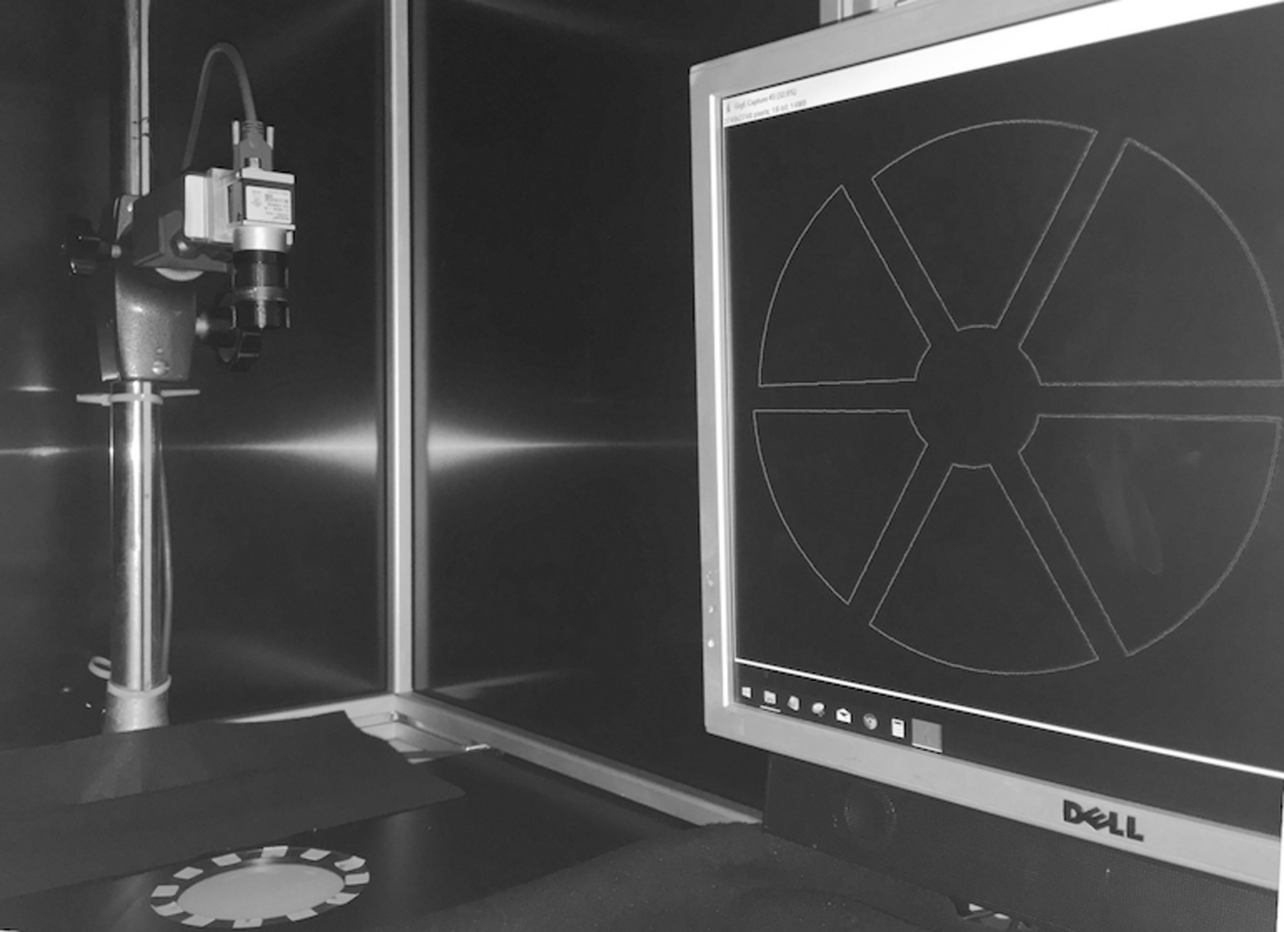
Camera setup. Grayscale digital camera for industrial image processing and LED backlight installed in a protective housing. PC screen displays predefined regions of interest for a Petri dish split into six segments.

A common LED powered photographer’s backlight for film and slide viewing is used for creating a transmissive illumination, which follows the suggestions published, e.g., by Chiang et al. [14]. The panel we used (M.WAY size A4 LED light panel, EAN 4710956777647) emits flicker free light at constant brightness over an area much larger than a Petri dish. The dish is kept in a fixed position at the center of the light panel by use of a jig and is illuminated uniformly. Fig 3 shows the final setup used.

The chosen camera comes complete with software (Basler pylon for Windows^®^), which allows parameter configuration and saving of images to disk. Recording parameters can be saved permanently in the camera allowing constant operating conditions without need of reconfiguration after startup. Optionally, direct camera control and image acquisition from within ImageJ is possible by using a software plugin made by Phase GmbH, Lübeck. This allows processing the recorded image immediately without previous saving to disk. Software was installed on a standard office PC equipped with 8 GB RAM and an Intel^®^ Core^TM^ i3 CPU running Windows^®^ 10, which was purchased for approximately €500.

### Image processing and colony enumeration

The camera used in our setup allows recording images with a brightness resolution of 8 or 16 bits per pixel. Both data formats can be processed in ImageJ. Changes of brightness and contrast in an 8-bit image will produce gaps in the brightness histogram, which can be avoided by performing all image manipulations on a 16-bit image. However, working in 8 bit returned results equivalent to the gold standard method (see below in “Results”) and thus all subsequent experiments were carried out on images of 8-bit grayscale depth.

Two assumptions were made in order to simplify processing of the recorded images:

- The size of the detected / counted objects is irrelevant, only their number needs to be determined exactly. This makes isolating the objects from the background of the image considerably easier. Instead of thresholding and segmentation, which is difficult for low contrast, confluent objects and proved unsatisfactory in preliminary tests, image processing focuses on identifying objects as features that cause a local brightness extreme. This approach can also detect multiple extremes in a neighborhood. Compared to complex strategies like fuzzy logic based detection [15] the solution suggested here is quite simple.
- Objects of interest (the CFUs) appear generally darker than the (local) background and each CFU possesses one darkest spot, which marks the center. This is in principle already achieved by the design of the illumination, although some post-processing of the image is required for noise suppression.

Taking the digital image and saving it to disk typically required less than ten seconds during our preliminary tests. Counting the colonies by image processing (sequential execution of one command after another) produced the result within less than ten seconds as well. For all comparisons discussed subsequently we thus generally assume that complete digital processing of a Petri dish by an operator with some routine requires about 20 s.

**Fig 4:**
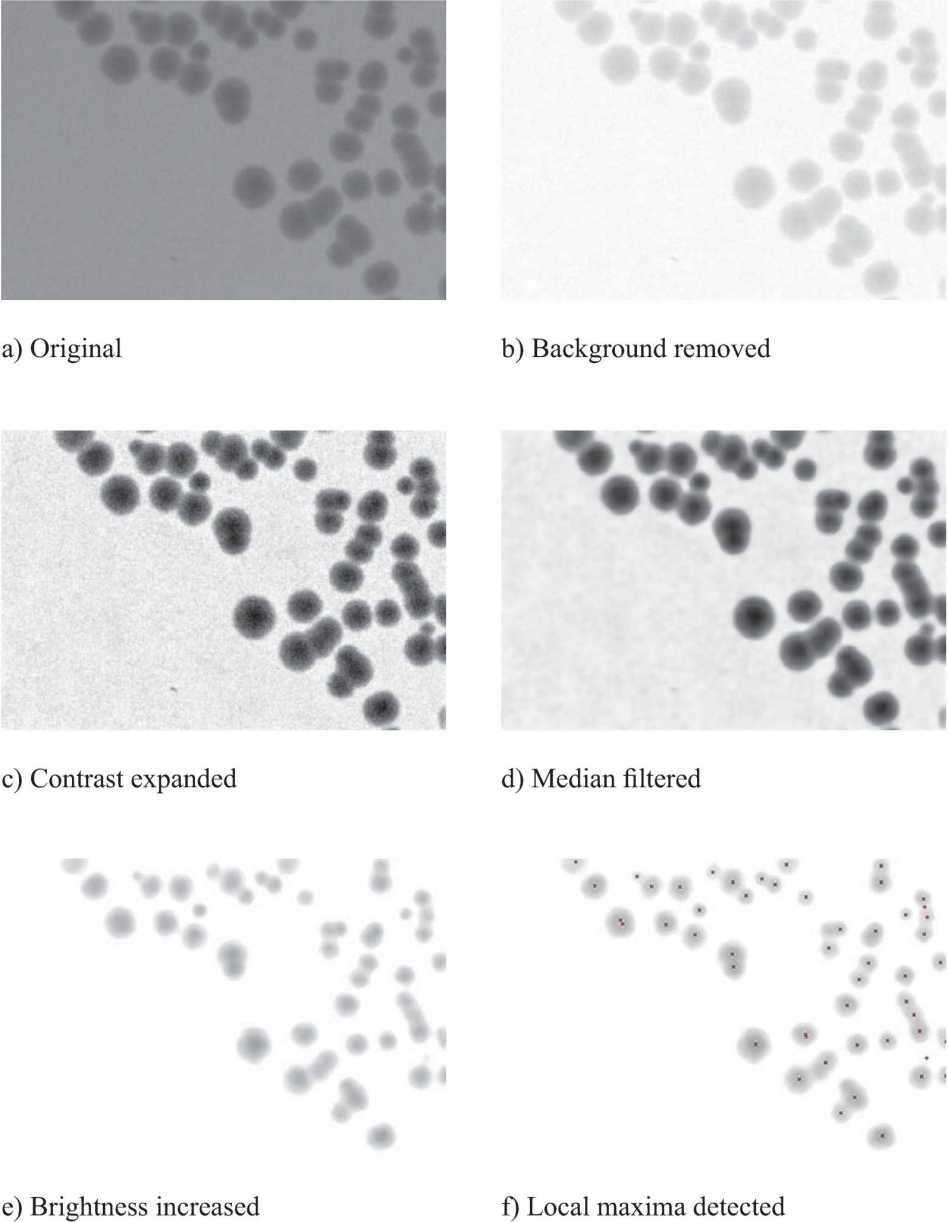
Sequence of processing steps. From top left to bottom right: a) original image, b) after background removal, c) after automatic contrast expansion, d) median filtered, e) after increasing image brightness (additional background removal step), f) with indicator spots for detected objects.

The processing steps that lead from the digital image to the CFU count are described one by one below. Fig 4 illustrates how the various steps affect the content of the region of interest.

As a first step the area to be processed and enumerated was selected in the recorded image. This is particularly important for the subsequent automatic contrast adjustment, which would be hampered by the presence of, e.g., marks or text written in black ink. Since the inoculation areas as well as the position of the Petri dish for imaging were fixed we could predefine regions of interest (ROIs), and no readjustment by the operator was required. In Fig 1 such regions of interest are shown, outlining the six segments of the plate.

In the following step the background of the selected region(s) of interest was smoothed by using ImageJ‘s *Subtract Background…* command (compare Fig 4A and 4B to see the effect). This function applies the *Rolling Ball* algorithm with a fixed radius, which should be larger than the largest object to be detected. The *E*. *coli* DH5α samples investigated here had been incubated for 24 h at 37°C, forming colonies smaller than 2 millimeters in diameter. We used a radius of 50 pixels for background subtraction, equivalent to 4 mm maximum object diameter (preservation of larger objects may require that the radius value is increased accordingly), to accommodate for different colony growth rates. Uniform results were achieved for varying background brightness (caused, e.g., by variable thickness of broth layer).

After background elimination we expanded image contrast by using ImageJ‘s *Brightness/Contrast* command in automatic mode. Applying this function spreads the gray values of the image across the available dynamic range, independent of the dynamic range in the original image (compare Fig 4B and 4C to see the effect). Background removal plus contrast expansion thus equalize images that may have initially differed in brightness. Proper contrast expansion requires that any gray value in the image represents either a part of the remaining background or, respectively, of the objects to be detected–no dark spots caused by impurities, smudging or writing must be present.

At this stage an image still contains residual noise in the background and within the objects to be detected and counted. In the next step thus noise was removed by median filtering. Median filtering suppresses noise in digital images much more efficiently than application of the Gauss or the average value filter while preserving edges and removing outliers (compare Fig 4C and 4D to see the effect). In our preliminary experiments we also tested other noise removal methods but, as expected, median filtering turned out to be the most suitable. Below the approximate size of the work area of the median filter objects get eliminated, which is sometimes regarded as a disadvantage [16]. This behavior, however, can also be used intentionally to exclude microcolonies. The chosen operating range (working radius of 6 pixels, equal to approximately 0.25 mm) corresponded to the minimum size of objects that would have been considered in manual counting.

Finally, the remaining background features had to be removed from the filtered image. Since the final counting step required a grayscale image thresholding could not be applied. Therefore a constant brightness value was added to all pixels of the image in order to lift the background, still containing low contrast artifacts, above the numerical brightness maximum (255 at 8 bit depth). Good results were achieved by raising image brightness by 135, so this value was generally applied. Compare Fig 4D and 4E to see the effect; the background area is uniformly white and the colonies appear lighter, but still with a grayscale structure.

After these processing steps the images showed, in front of a uniform white background, objects which had originally been larger than the minimal size defined by the median filter. The CFU number was then determined by counting local brightness minima in the image using ImageJ’s *Find Maxima…* command (using the *Light background* option). Fig 4F shows the result: In this example, the detected center points were copied into Fig 4E.

The image processing method described above eliminated background artifacts and ensured that a single local density maximum represented the center of a CFU. Still, clustered colonies show multiple local density maxima, i.e., multiple CFUs may be recognized up to a certain degree of overlapping.

**Fig 5:**
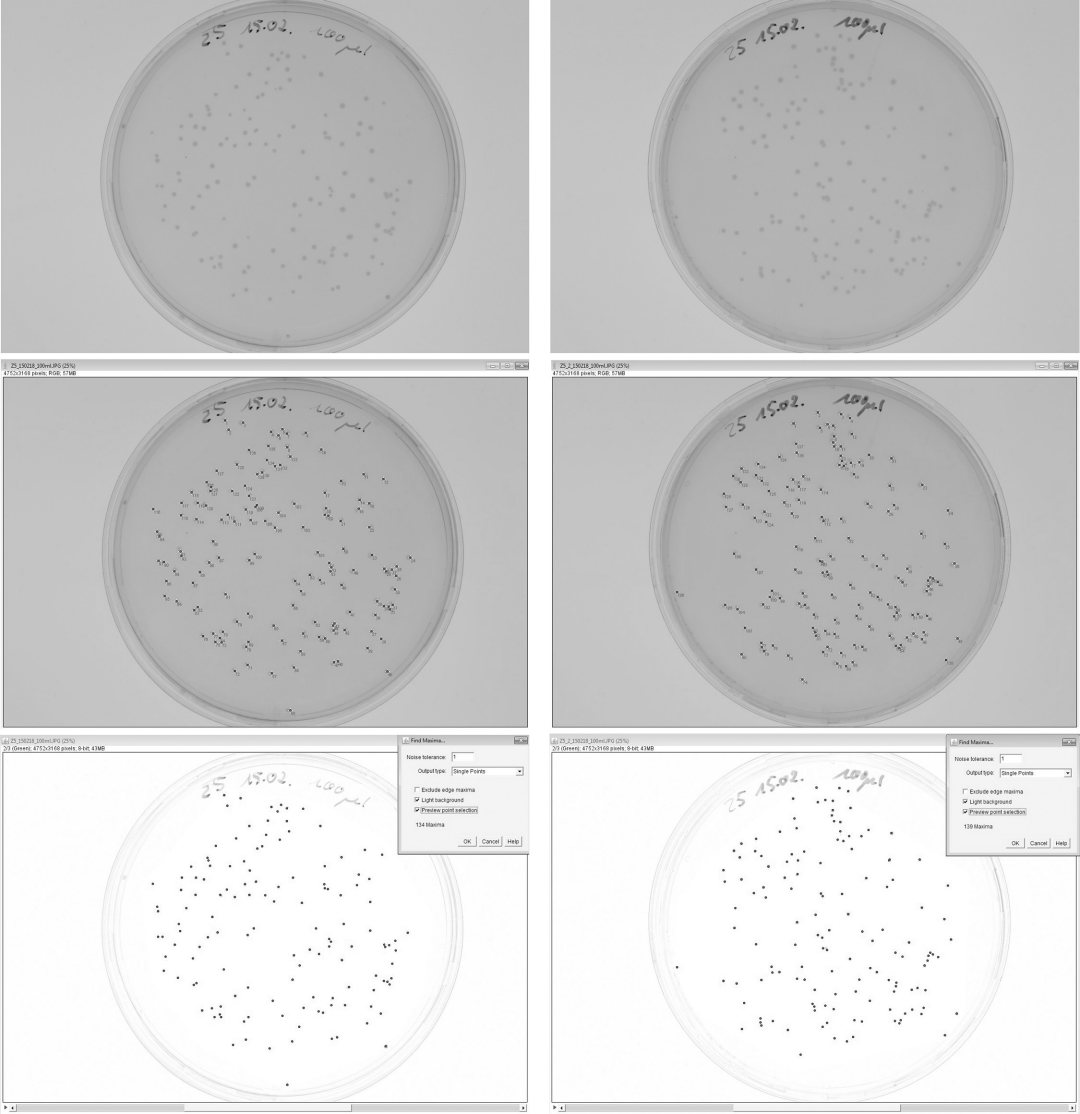
Manual and automatic enumeration of two non-segmented plates. Top row: original images, middle row: enumeration result of point/click method (counts: 136, 137), bottom row: enumeration by image analysis (counts: 134, 139). Samples inoculated with *E*. *coli* DH5α.

Fig 5 shows two examples of non-segmented plates from a series of images recorded during the test phase of the image processing sequence at approximately 600 dpi resolution, using a digital SLR camera. The images were first enumerated manually on-screen by pointing/clicking in order to obtain the "gold standard" reference count; the results were 136 (left plate) and 137 (right plate). The respective automatic counts (obtained by processing the image as described below) were 134 and 139. Manual enumeration of plates like those in Fig 4 required time in the order of magnitude of one minute per plate even though the diameter ratio of largest to smallest colony was approximately 4, facilitating manual counting. For more details see below in “Results”.

**Fig 6:**
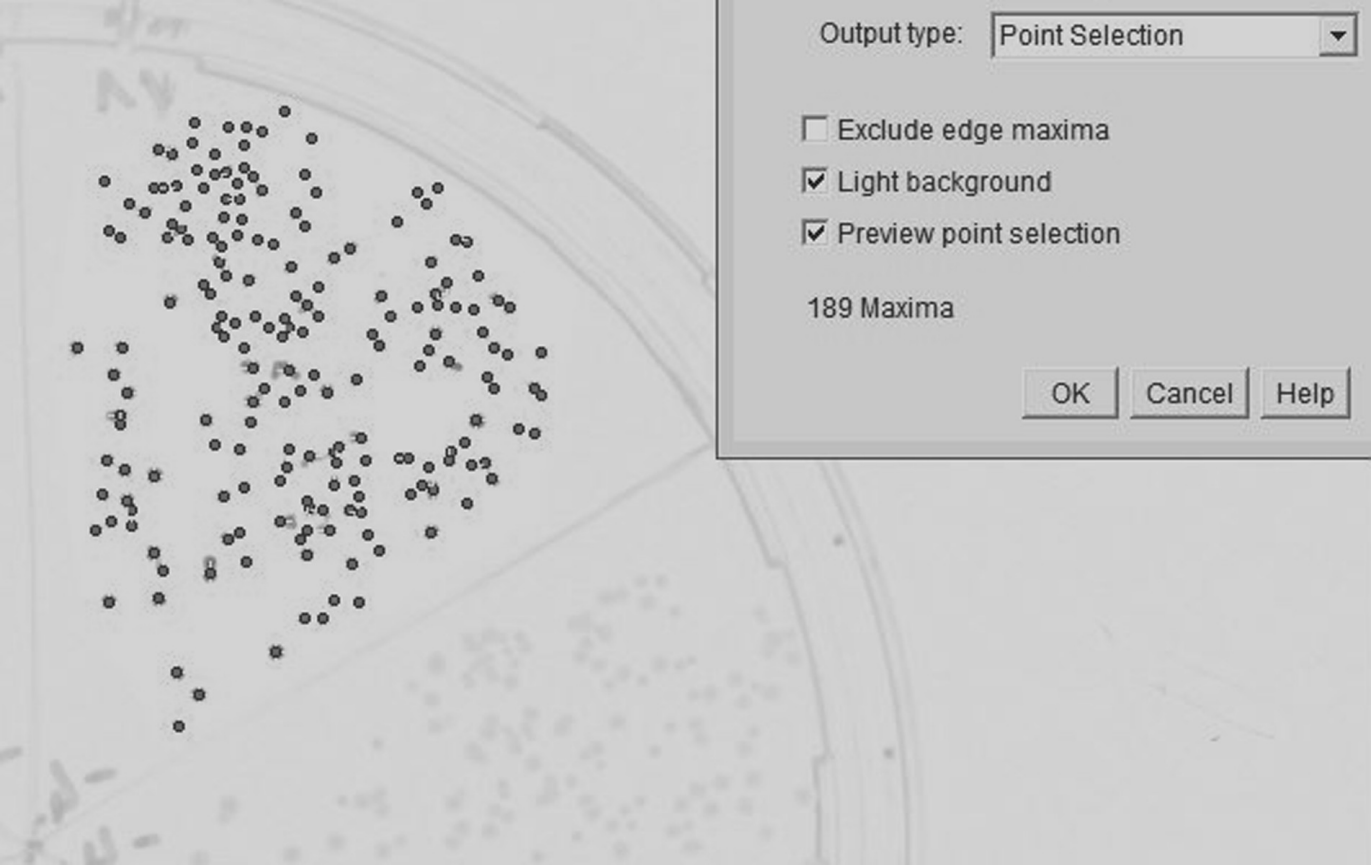
Example enumerated segment. CFU count of a densely populated 1/6-plate segment, determined by image processing.

If the agar plate is segmented counting becomes more challenging, as shown exemplarily in Fig 2 where approximately 300 colonies mark the practical limit of manual counting. Automated counting by image processing, however, appeared to be still feasible for such high CFU numbers. Fig 6 shows an automatically counted segment with almost 200 colonies.

The above-described method resulted in better recognition of confluent colonies than methods employing binary thresholding and segmentation (using, e.g., watershed separation), which we tried as alternatives. Advanced shape specific filtering, e.g., Hough transform, might lead to equivalent or better results [17], but this would be beyond the scope of the simple procedure we intended to create.

In order to compare the automatic enumeration systematically with the previous counting method (marking CFUs with a pen on the dish lid) as well as with a reference standard incubated Petri dishes from multiple series of experiments were then enumerated in three different ways:

- Semi-manual counting by clicking on the colonies as viewed in a digital image of the Petri dish on a computer screen, in 1:1 representation (screen pixel: image pixel). Numbers were recorded by software (ImageJ offers specific functions for point click counting of objects). This represented the "gold standard" and was done by three skilled persons.
- Manual counting by marking colonies on the lid of the Petri dish with a felt marker–the persons performing the task kept the count in memory until finishing one plate (or segment) or took notes. Three skilled persons enumerated the plates.
- Automatic enumeration using a macro (predefined set of commands) in ImageJ. This macro comprised the digital image processing steps as described above. All images were processed using the same parameter set. Automatic enumeration was not repeated; since all parameters were predefined neither count nor processing time varied (this had previously been established by repeated enumeration of several Petri dishes).

For each enumerated plate the CFU count as well as the time required were noted. Preparation times are not considered here–if large numbers of plates are processed the time required for daily startup of the camera system is insignificant.

## Results

The above-described method of enumeration by image processing was developed over a period of several months, using sample Petri dishes as they became available from ongoing experiments. Once the method had been established and the camera setup had been finalized, additional experiments were carried out on a number of plates in order to determine count repeatability and counting time in comparison with traditional methods. About half the number of plates were inoculated only with one sample on the full area, while the others were split in six segments (two segments respectively for three dilution levels). The results are discussed below.

Table 1 shows numerical values of the count ratios, i.e., the CFU numbers determined by automatic (image analysis) and manual (pen marking) enumeration in relation to the gold standard count (on screen click marking on a high resolution image). The table also lists the number of colonies per plate and the counting rates achieved by manual and gold standard counting. In the case that ROIs were enumerated that represented a 1/6-segment of a plate, the respective CFU count was multiplied by 6 in order to make the results comparable with enumeration of complete plates. Counts and count rates are averages of three enumerations each.

**Table 1:** Overview of enumeration results. CFU numbers determined by manual, automatic and gold standard counting of plates. Count ratios of automatic and manual enumeration in relation to the gold standard method. Count rates of manual and gold standard enumeration. Counts and counting rates in rows 1–25 are based on three enumerations each.

#	Counts/s	Count	Counts/s	Count	Count	Count ratio	Count ratio	Segments
	Gold	Gold	Manual	Manual	Automatic	Auto/Gold	Manual/Gold	/plate
	•	•	•	•	•	•	•	•
1	1.14 ±0.03	174.0 ±0.0	1.87 ±0.25	168.0 ±6.0	174	1.00	0.97	6
2	1.31 ±0.08	210.0 ±0.0	1.73 ±0.17	208.0 ±3.5	198	0.94	0.99	6
3	1.16 ±0.21	176.0 ±3.5	1.88 ±0.47	188.0 ±13.9	198	1.13	1.07	6
4	1.52 ±0.03	234.0 ±0.0	2.09 ±0.08	196.0 ±12.5	210	0.90	0.84	6
5	0.72 ±0.11	754.7 ±3.8	1.52 ±0.27	714.3 ±22.9	733	0.97	0.95	1
6	1.34 ±0.19	612.0 ±19.0	1.65 ±0.35	578.0 ±15.0	583	0.95	0.94	1
7	1.38 ±0.09	579.0 ±3.5	1.63 ±0.23	547.7 ±26.3	566	0.91	0.88	1
8	1.35 ±0.17	67.3 ±2.3	1.56 ±0.63	63.0 ±5.2	70	1.04	0.94	1
9	1.17 ±0.19	14.0 ±0.0	1.91 ±0.66	14.0 ±0.0	12	0.86	1.00	1
10	0.82 ±0.28	316.0 ±3.5	2.00 ±0.38	284.0 ±9.2	318	1.01	0.90	6
11	1.03 ±0.06	396.0 ±0.0	2.67 ±0.51	304.0 ±12.5	396	1.00	0.77	6
12	0.99 ±0.07	288.0 ±0.0	1.52 ±0.08	276.0 ±6.0	288	1.00	0.96	6
13	0.80 ±0.07	418.0 ±3.3	3.21 ±0.67	404.0 ±12.5	414	0.99	0.97	6
14	0.80 ±0.16	456.0 ±0.0	2.49 ±0.33	364.0 ±9.2	408	0.89	0.80	6
15	0.97 ±0.10	510.0 ±0.0	2.57 ±0.60	442.0 ±15.1	468	0.92	0.87	6
16	1.03 ±0.06	81.3 ±1.1	2.19 ±0.60	76.7 ±2.5	80	0.98	0.94	1
17	1.08 ±0.31	149.7 ±1.5	1.84 ±0.46	141.0 ±8.2	150	1.00	0.94	1
18	0.75 ±0.11	288.0 ±0.9	1.83 ±0.22	272.7 ±3.8	289	1.00	0.95	1
19	0.87 ±0.26	246.7 ±2.0	1.72 ±0.08	210.3 ±23.2	235	0.95	0.85	1
20	1.28 ±0.16	615.3 ±18.5	1.63 ±0.29	564.0 ±7.9	593	0.96	0.92	1
21	1.50 ±0.28	555.0 ±10.5	1.40 ±0.27	459.0 ±149	547	0.99	0.83	1
22	1.01 ±0.11	300.0 ±0.0	1.78 ±0.24	288.0 ±6.0	300	1.00	0.96	6
23	1.14 ±0.30	300.0 ±0.0	1.35 ±0.18	276.0 ±6.0	300	1.00	0.92	6
24	1.20 ±0.0	230.0 ±3.5	1.48 ±0.0	204.0 ±10.4	210	0.91	0.89	6
25	1.21 ±0.12	284.0 ±3.4	2.01 ±0.05	274.0 ±3.5	270	0.95	0.96	6
	•	•	•	•	•	•	•	•
	Average	•	Average	•	•	Average	Average	•
	1.1/s	•	1.9/s	•	•	0.97	0.92	•

**Fig 7:**
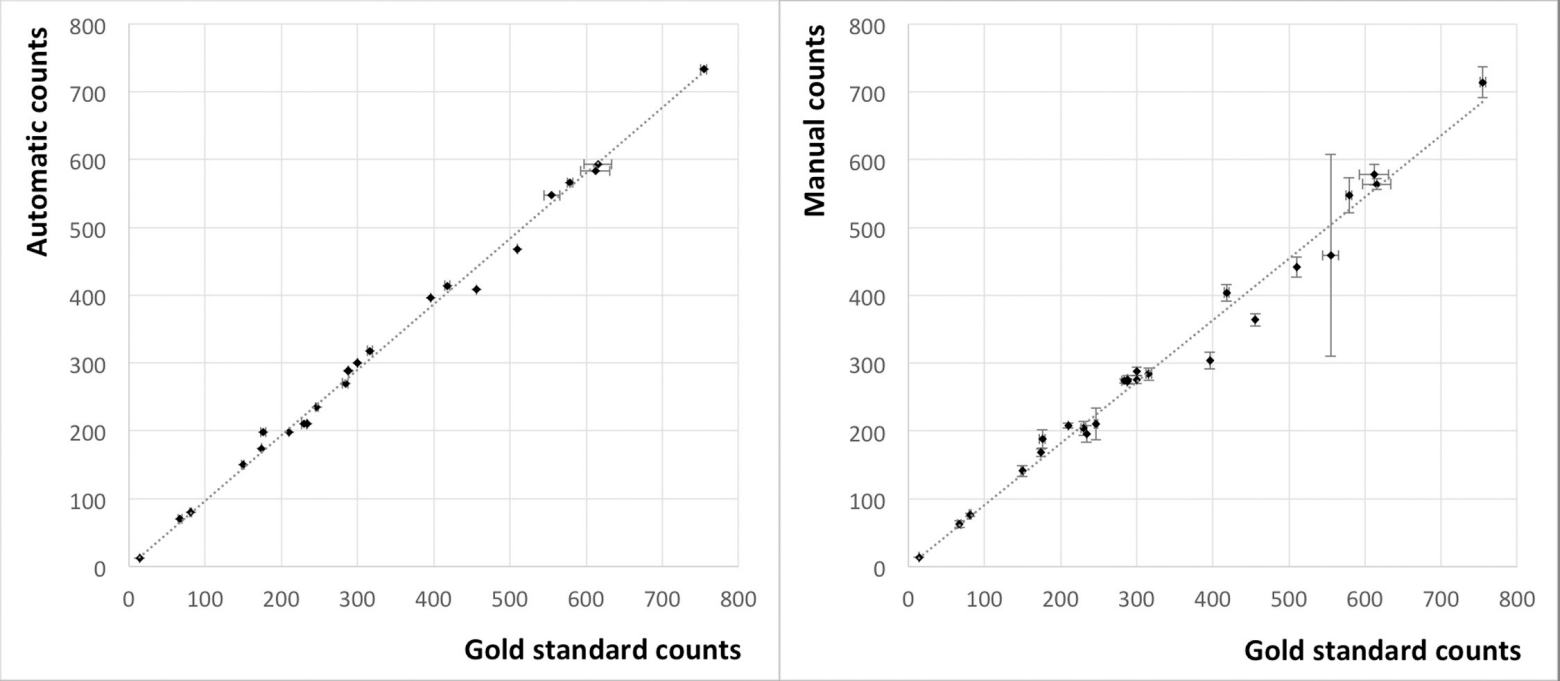
Method correlation. CFU counts for 25 samples of plates inoculated with *E*. *coli* DH5α. Left diagram: automatic vs. gold standard counts, right diagram: manual vs. gold standard counts. Error bars in x and y directions represent the standard deviation of three enumerations.

Fig 7 shows results of automatic and manual counting, plotted against the gold standard method, respectively. Horizontal error bars indicate the standard deviation of the three gold standard repetitions. Vertical error bars in the right diagram indicate the standard deviation of manual counting. Automatic enumeration (left diagram) always returns the same result for a given sample, i.e., the y error is zero for all automatic counts. The gold standard method also returned three identical counts (i.e., zero x error) for 10 out of the 25 samples.

A correlation analysis was carried out, assuming that for small CFU numbers the methods must match (i.e., forcing the regression lines through the origin as drawn in Fig 7). This restraint, along with the fact that the standard deviation for some of the individual data points was zero in either one or both directions, turned out to be a difficulty for linear regression including error analysis. Instead of using the individual x and y standard deviations of each data point in the analysis we had to assign average percentaged errors. The gold standard counts on average had a variation coefficient of 1%, which was assigned as standard x error to the gold standard values. The manual counts on average had a variation coefficient of 5%, which was assigned as standard y error to the manual values. A fictitious variation coefficient of 1‰ was assigned as standard y error to the automatic values. Solutions could then be calculated using Levenberg-Marquardt’s algorithm.

For manual vs. gold standard a slope of 0.913 with a 95% confidence interval of *CI*_95%_ = (0.896, 0.93) was determined. Automatic vs. gold standard correlated with a slope of 0.976 and *CI*_95%_ = (0.972, 0.98). For the experiments in this study automatic counting results would thus be equivalent to the gold standard if multiplied by 1/0.976 = 1.025, and manual results would be equivalent if corrected by a factor of 1.095.

**Fig 8:**
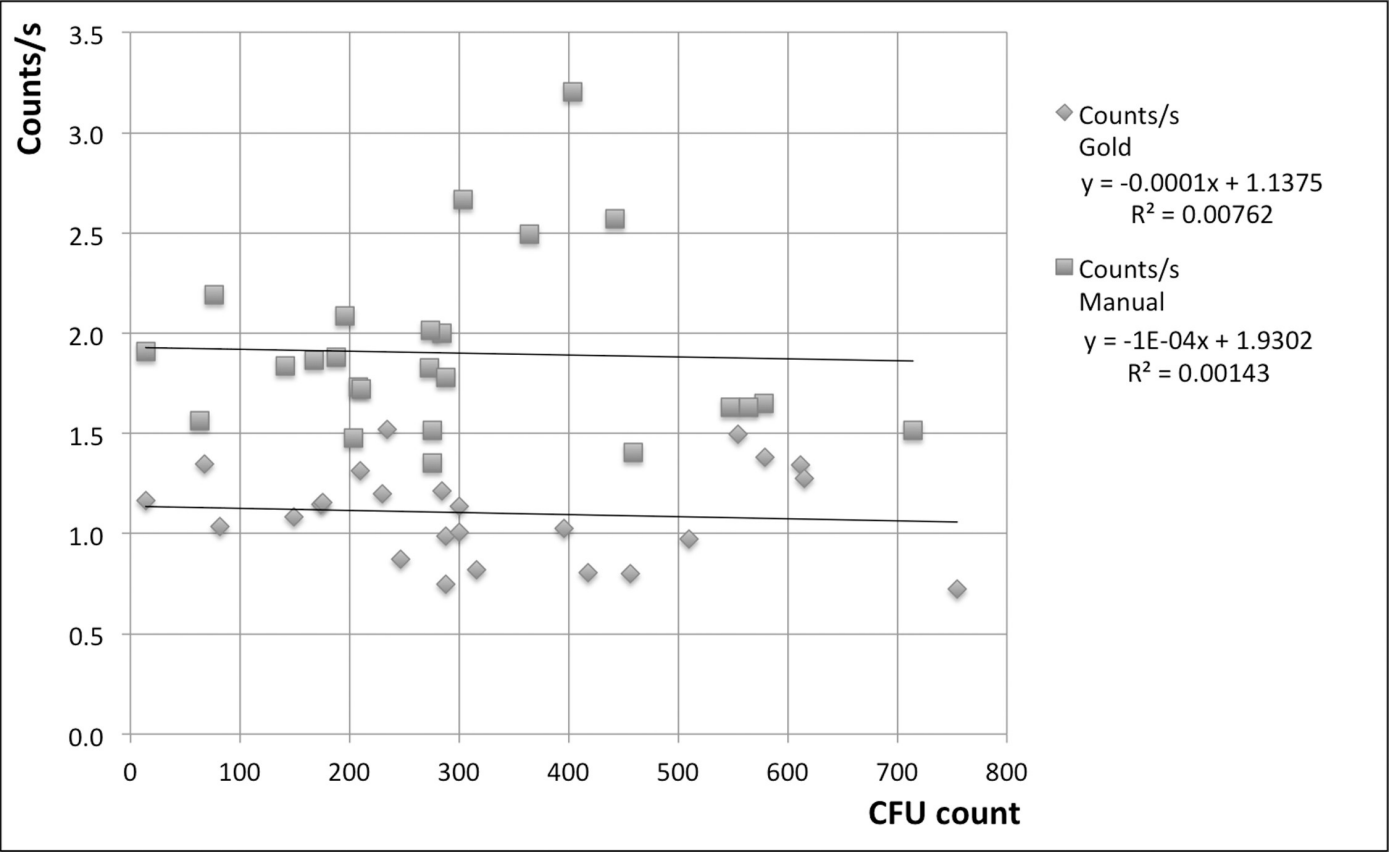
Counting rates. Rates (markings per second) for manual and gold standard CFU enumeration achieved for all plates enumerated for this study. All test were carried out on plates inoculated with *E*. *coli* DH5α. Each data point represents the average of three enumerations.

Fig 8 shows the observed counting rates (markings per second) for manual and gold standard enumeration. Correlation coefficient values close to zero indicate that there is no relation between the counting rates and the CFU number. One should keep in mind, however, that the plates enumerated here were produced as part of a small, specific experiment and the persons who enumerated them did not experience fatigue in the same way as they might in case of repetitive enumeration of large numbers of plates. Even so, the data for the manual counting rate show more scatter compared to the gold standard rate.

**Fig 9:**
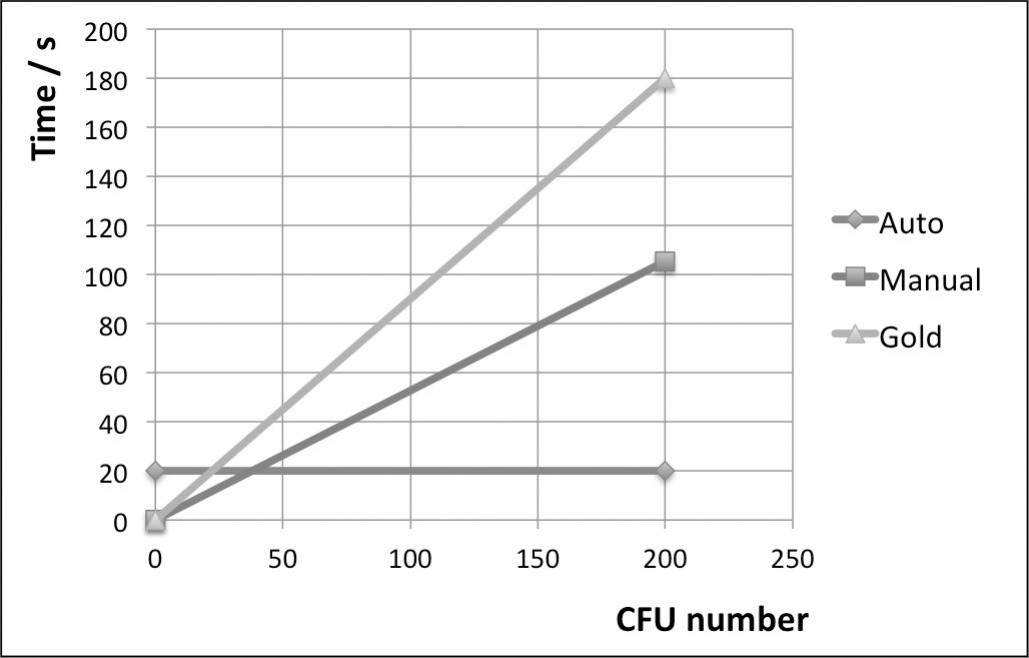
Required counting time as a function of colony count. Lines based on constant enumeration time of 20 s for automatic method, and count rates for manual and gold standard counting as shown in Fig 8 and Table 1.

The counting rate for manual counting was 1.9/s on average, while the gold standard method was slower at 1.1/s. A counting rate for the automatic method can't be given, since the process always requires the same 20 s irrespective of the number of colonies, but as an example we can compare times for a plate containing 100 colonies: The gold standard method would require approximately 91 s while manual counting takes 53 s. Fig 9 illustrates the advantage gained by applying the automatic method for increasing CFU counts.

## Discussion

The automatic method returned counting results that were on average only slightly below the gold standard count, performing better in this respect than the manual method. The linear regression against the gold standard also returned a smaller 95% confidence interval for automatic counting compared to manual counting. Automatic counting time is independent of the number of colonies, while gold standard and manual counting times grow proportionately with the CFU count. Automatic counts are independent of operator's skill and fatigue, which makes the method particularly attractive for laboratories that need to enumerate large numbers of the same type of samples recurringly. However, the output strongly depends on the chosen values of various parameters that control the image processing operations. When setting up an image processing system for the first time, or when a new type of samples is introduced, an initial series of tests involving automatic and gold standard enumeration is thus required. One should determine a parameter set for the automatic method that leads to a good match, and the correction factor to achieve equivalence between gold standard and automatically determined results. From our point of view it would also be good practice to repeat such comparisons at intervals.

In this study we examined *E*. *coli* DH5α, which forms circular colonies and has a fairly uniform growth rate–at the end of the incubation period the colonies were between 0.5 and 4 mm in diameter. The results (and the values of the command parameters used) should be transferable to the enumeration of other microorganisms, which behave similarly.

**Fig 10:**
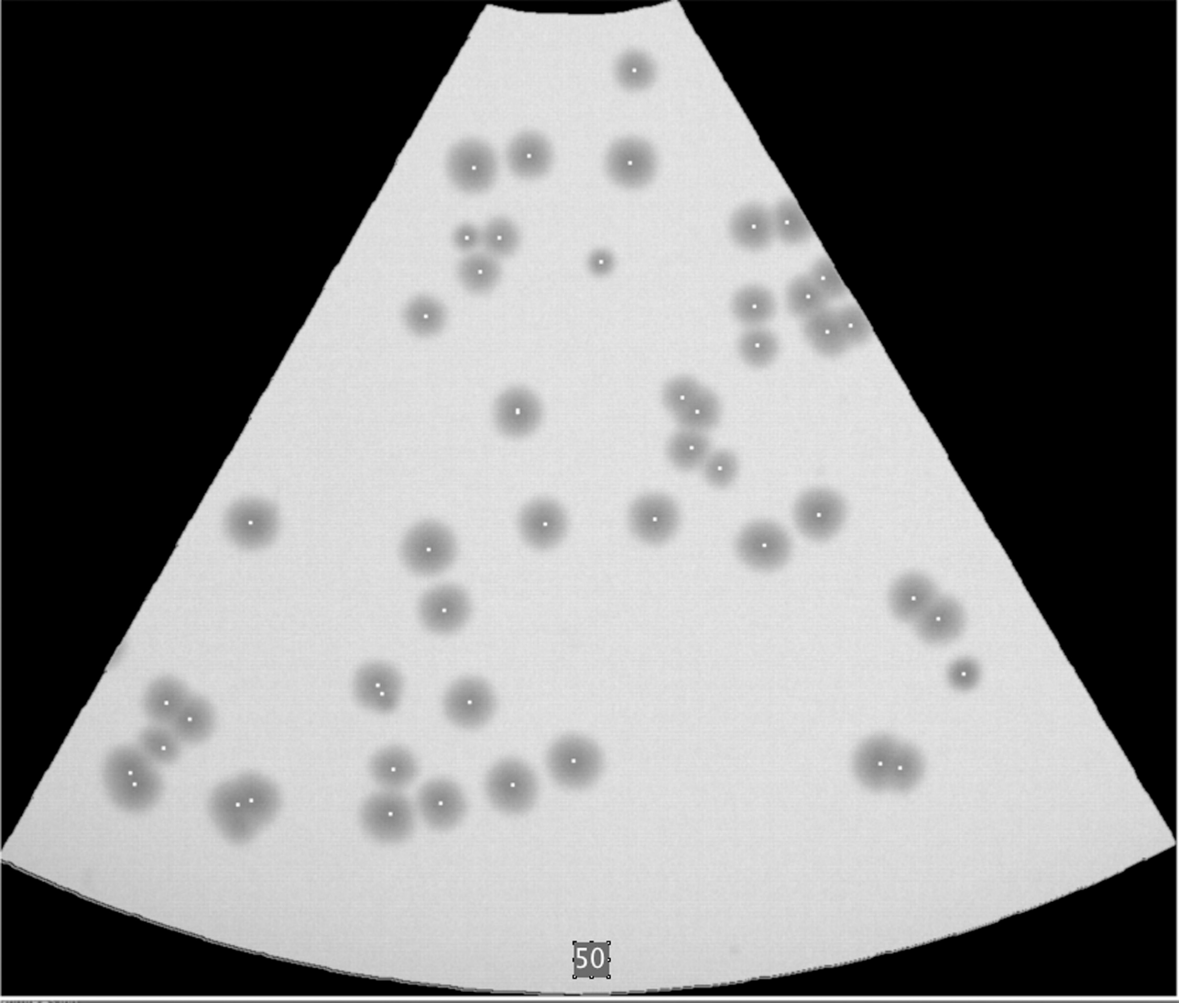
Example for visualization of results. Plate segment processed and detection results copied as white spots into the original image. Numerical counting result typed to a text field.

It is also recommendable to carry out a final visual check of the detection performance by comparing the original image and the result of ImageJ's *Find Maxima* operation for densely populated plates (e.g., more than 200 colonies for a complete dish). The *Single Points_Output* option may be applied to deliver single point marks of the CFU centers, which can be made well visible by one or two steps of dilatation. Visual checking is then greatly facilitated by either creating an overlay of original image and detected points map, or by combining both to an image "stack" and cycling between the two quickly. Fig 10 shows an example of a plate segment with dots marking the centers of detected CFUs.

Another very useful feature of ImageJ is its ability to type results as text directly into an image, as shown in Fig 10. It is thus possible to create an automated command sequence or macro [18], which does not just count the CFUs but also creates a result image containing detected CFU centers as well as the numeric count for each region of interest. The saved data will then contain the original image, which may be re-evaluated again, as well as detailed results. It is also possible to append a documentation of all processing steps carried out, and the respective parameter values, to each image.

Our expectations regarding the potential benefits to be earned from a comparatively small investment in image processing hardware and software were fulfilled. The equipment described here is of industrial quality and can thus be expected to have an extended life span. The automatic enumeration

- returns results which are closer to the gold standard than the previously used manual method,
- saves a considerable amount of time in the laboratory,
- improves our quality management because plate images are stored, so that counting results can be proofed and reproduced,
- was implemented with purchase cost for equipment even below our initial budget.

## Conclusion

We describe the development of an experimental setup and data processing steps for automated imaging and counting of *E*. *coli* DH5α colonies grown on 110 mm Petri dishes. The hardware used is easily available and, at less than €2,000, purchase cost is only a small fraction of that of a comparable commercial system. Image processing was done using the public domain software package ImageJ. Processing steps for fast and reliable detection of *E*.*coli* DH5α colonies are described in detail, allowing readers to adapt the method to their needs by reproducing–and, if necessary, adjusting–these processing steps. Fast evaluation of large numbers of samples can be achieved with the equipment used here by creating an automated command sequence. The resulting system can greatly reduce the time required for CFU enumeration compared to manual counting. It allows CFU counting independent of subjective influence and creation of a database of samples and enumeration results.

## Supporting information

S1 Data (XLSX)

S1 File (PDF)

S2 File (PDF)

S3 File (PDF)

S4 File (PDF)

## References

[1] DIN 10192–5. Microbiological analysis of milk—Colony count—Part 5: Spatula method.

[2] Brugger SD, Baumberger C, Jost M, Jenni W, Brugger U, Mühlemann K. Automated Counting of Bacterial Colony Forming Units on Agar Plates. PLoS ONE 2012; 7(3): e33695 10.1371/journal.pone.0033695 22448267 PMC3308999

[3] Sutton S. Accuracy of Plate Counts. Journal of Validation Technology (Summer 2011): 42–46.

[4] Breed RS, Dotterrer WD. The Number of Colonies Allowable on Satisfactory Agar Plates. J Bacteriol 1916; 1(3): 321–331. 16558698 10.1128/jb.1.3.321-331.1916 PMC378655

[5] Zhang C, Chen W. An effective and robust method for automatic bacterial colony enumeration. International Conference on Semantic Computing 2007:581–8.

[6] Putman M, Burton R, Nahm MH. Simplified Method to Automatically Count Bacterial Colony Forming Unit. J Immunol Methods 2005;302:99–102. 10.1016/j.jim.2005.05.003 16002082

[7] Zhang C, Chen W, Liu W and Chen C. An Automated Bacterial Colony Counting System. IEEE International Conference on Sensor Networks, Ubiquitous, and Trustworthy Computing 2008:233–240.

[8] Young LM, Rieman DJ, Walden L, Motz VA. In search of a counter you can count on: relative efficacy of human visual and automated colony counting. Letters in Applied Microbiology 2018;66:188–193. 10.1111/lam.12851 29341168

[9] Sethi H, Yadav S. Bacterial Colony Counter: Manual vs Automatic. Engineering Science and Technology 2012;2(1):42–4

[10] Hwang JC. NIST's Integrated Colony Enumerator (NICE). 2010. Available from: https://www.nist.gov/services-resources/software/nists-integrated-colony-enumerator-nice

[11] Clarke ML, Burton RL, Hill AN, Litorja M, Nahm MH, Hwang J. Low-cost, high-throughput, automated counting of bacterial colonies. Cytometry A. 2010 8;77(8):790–7 10.1002/cyto.a.20864 20140968 PMC2909336

[12] Geissmann Q. OpenCFU, a New Free and Open-Source Software to Count Cell Colonies and Other Circular Objects. PloS ONE 2013;8(2):e54072 10.1371/journal.pone.0054072 23457446 PMC3574151

[13] Basler. Technical Specification BASLER LENS C125-1620-5M, Document Number: DG001468. 2016. Available from: https://www.baslerweb.com/en/sales-support/downloads/document-downloads/

[14] Chiang PJ, Tseng MJ, He ZS, Li CH. Automated counting of bacterial colonies by image analysis. J of Microbiological Methods 2015;108:74–82. 10.1016/j.mimet.2014.11.009 25451456

[15] Marotz J, Lübbert C, Eisenbeiß W. Effective object recognition for automated counting of colonies in Petri dishes (automated colony counting). Computer Methods and Programs in Biomedicine 2001;66:183–198. 10.1016/s0169-2607(00)00128-0 11551392

[16] Kaur G, Sethi P. A Novel Methodology for Automatic Bacterial Colony Counter. International Journal of Computer Applications 2012;49(15):21–6.

[17] Barber PR, Vojnovic B, Kelly J, Mayes CR, Boulton P, Woodcock M, et al Automatic counting of mammalian cell colonies. J Physics in Medicine and Biology 2001;46(1):63–76. 10.1088/0031-9155/46/1/305 11197679

[18] Mutterer J, Rasband W. The ImageJ Macro Language. 2012. Available from: https://imagej.nih.gov/ij/docs/macro_reference_guide.pdf

