# Supplementary material for: Experimental setup and image processing method for automatic enumeration of bacterial colonies on agar plates
Source: PLoS One. 2020 Jun 24;15(6):e0232869. doi: 10.1371/journal.pone.0232869 (PMC7313745; doi:10.1371/journal.pone.0232869)
Supplement: S1 File — (PDF) [file pone.0232869.s002.pdf]

LENS QUALITY: MTF, RESOLUTION & CONTRAST

Lens quality is more important now than ever, due to the ever-increasing number of megapixels found in today's digital cameras. Frequently, the resolution of your digital photos is actually limited by the camera's lens — and not by the resolution of the camera itself. However, deciphering MTF charts and comparing the resolution of different lenses can be a science unto itself. This tutorial gives an overview of the fundamental concepts and terms used for assessing lens quality. At the very least, hopefully it will cause you to think twice about what's important when purchasing your next digital camera or lens.

Download
surprised

RESOLUTION & CONTRAST

Everyone is likely to be familiar with the concept of image resolution, but unfortunately, too much emphasis is often placed on this single metric. Resolution only describes how much detail a lens is capable of capturing — and not necessarily the quality of the detail that *is* captured. Other factors therefore often contribute much more to our perception of the quality and sharpness of a digital image.

To understand this, let's take a look at what happens to an image when it passes through a camera lens and is recorded at the camera's sensor. To make things simple, we'll use images composed of alternating black and white lines ("line pairs"). Beyond the resolution of your lens, these lines are of course no longer distinguishable:

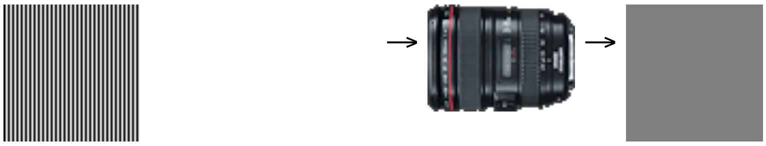

High Resolution Line Pairs Lens Unresolved Line Pairs

Example of line pairs which are smaller than the resolution of a camera lens.

However, something that's probably less well understood is what happens to other, thicker lines. Even though they're still resolved, these progressively deteriorate in both contrast and edge clarity (see sharpness: resolution and acutance) as they become finer:

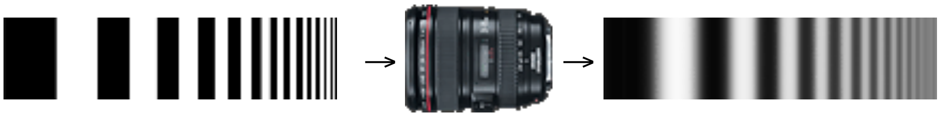

Progressively Finer Lines Lens Progressively Less Contrast & Edge Definition

For two lenses with the same resolution, the apparent quality of the image will therefore be mostly determined by how well each lens preserves contrast as these lines become progressively narrower. However, in order to make a fair comparison between lenses we need to establish a way to quantify this loss in image quality...

MTF: MODULATION TRANSFER FUNCTION

A Modulation Transfer Function (MTF) quantifies how well a subject's regional brightness variations are preserved when they pass through a camera lens. The example below illustrates an MTF curve for a perfect* lens:

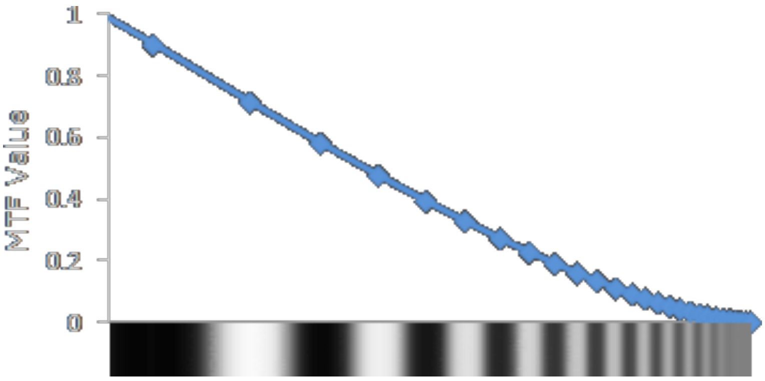

Increasing Line Pair Frequency →

Far right = Maximum Resolution / Diffraction Limit.

Note: The spacing between black and white lines has been exaggerated to improve visibility.
MTF curve assumes a circular aperture; other aperture shapes will produce slightly different results.

*A perfect lens is one whose detail is limited only by diffraction.
See tutorial on diffraction in photography for a background on this topic.

An MTF of 1.0 represents perfect contrast preservation, whereas values less than this mean that more and more contrast is being lost — until an MTF of 0, where line pairs can no longer be distinguished at all.

This resolution limit is an unavoidable barrier with any lens; it only depends on the camera lens aperture and is unrelated to the number of megapixels. The figure below compares a perfect lens to two real-world examples:

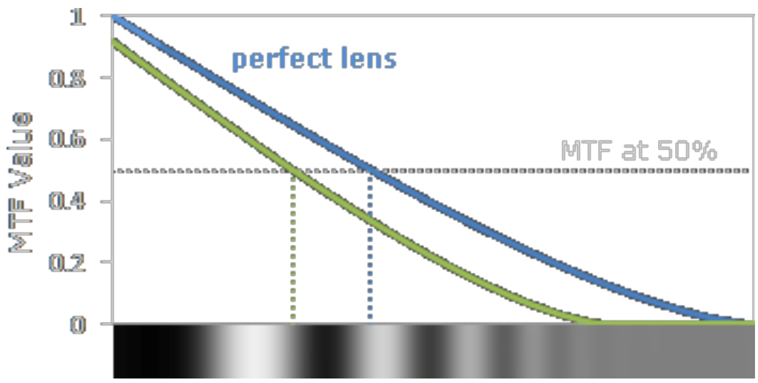

Increasing Line Pair Frequency →

Very High Quality Camera Lens (close to the diffraction limit) | Low Quality Camera Lens (far from the diffraction limit)

Comparison between an ideal diffraction-limited lens (blue line) and real-world camera lenses.
The line pair illustration below the graph does not apply to the perfect lens.
Move your mouse over each of the labels to see how high and low quality lenses often differ.

The blue line above represents the MTF curve for a perfect "diffraction limited" lens. No real-world lens is limited only by diffraction, although high-end camera lenses can get much closer to this limit than lower quality lenses.

Line pairs are often described in terms of their frequency: the number of lines which fit within a given unit length. This frequency is therefore usually expressed in terms of "LP/mm" — the number of line pairs (LP) that are concentrated into a millimeter (mm). Alternatively, sometimes this frequency is instead expressed in terms of line widths (LW), where two LW's equals one LP.

The highest line frequency that a lens can reproduce without losing more than 50% of the MTF ("MTF-50") is an important number, because it correlates well with our perception of sharpness. A high-end lens with an MTF-50 of 50 LP/mm will appear far sharper than a lower quality lens with an MTF-50 of 20 LP/mm, for example (presuming that these are used on the same camera and at the same aperture; more on this later).

However, the above MTF versus frequency chart is not normally how lenses are compared. Knowing just the (i) maximum resolution and (ii) MTF at perhaps two different line frequencies is usually more than enough information. What often matters more is knowing how the MTF changes depending on the distance from the center of your image.

The MTF is usually measured along a line leading out from the center of the image and into a far corner, for a fixed line frequency (usually 10-30 LP/mm). These lines can either be parallel to the direction leading away from the center (sagittal) or perpendicular to this direction (meridional). The example below shows how these lines might be measured and shown on an MTF chart for a full frame 35mm camera:

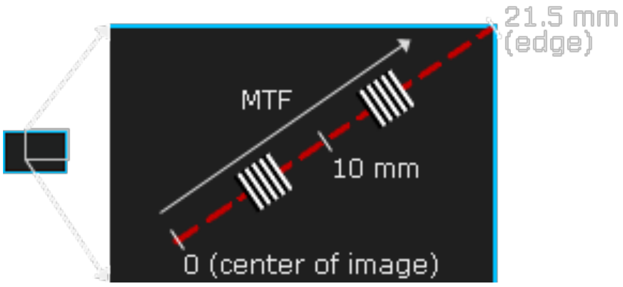

Meridional (Circular) Line Pairs
Sagittal (Radial) Line Pairs
→

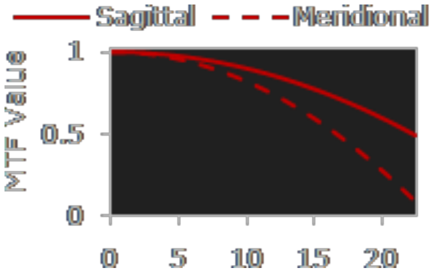

Distance From Center [mm]

Detail at the center of an image will virtually always have the highest MTF, and positions farther from the center will often have progressively lower MTF values. This is why the corners of camera lenses are virtually always the softest and lowest quality portion of your photos. We'll discuss why the sagittal and meridional lines diverge later.

HOW TO READ AN MTF CHART

Now we can finally put all of the above concepts into practice by comparing the properties of a zoom lens with a prime lens:

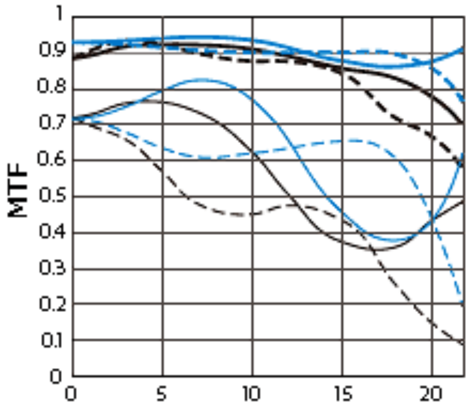

Canon 16-35mm f/2.8L II Lens (zoom set at 35mm)

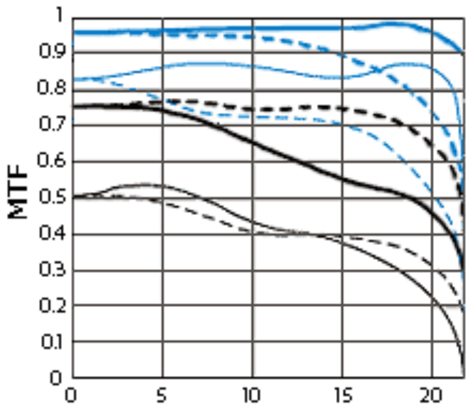

Canon 35mm f/1.4L Prime Lens

On the vertical axis, we have the MTF value from before, with 1.0 representing perfect reproduction of line pairs, and 0 representing line pairs that are no longer distinguished from each other. On the horizontal axis, we have the distance from the center of the image, with 21.6 mm being the far corner on a 35 mm camera. For a 1.6X cropped sensor, you can ignore everything beyond 13.5 mm. Furthermore, anything beyond about 18 mm with a full frame sensor will only be visible in the extreme corners of the photo:

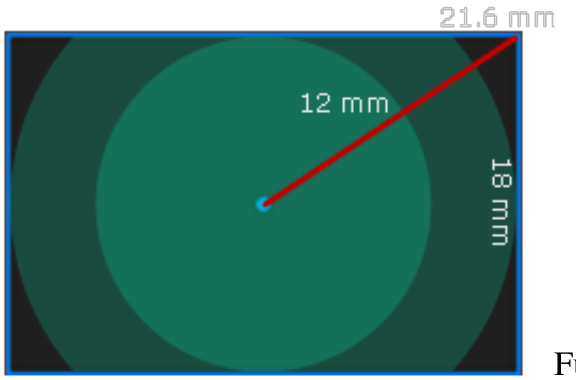

Full Frame 35 mm Sensor

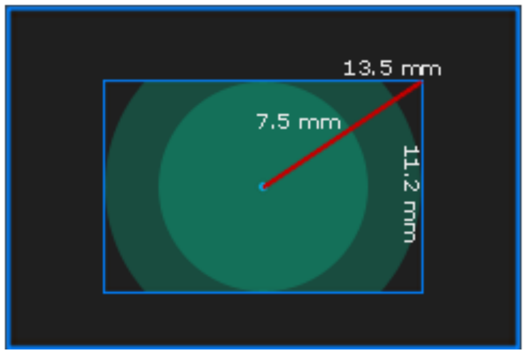

1.6X Cropped Sensor

Note: For a 1.5X sensor, the far corner is at 14.2 mm, and the far edge is at 11.9 mm.
See the tutorial on [digital camera sensor sizes](#) for more on how these affect image quality.

All of the different looking lines in the above MTF charts can at first be overwhelming; the key is to look at them individually. Each line represents a separate MTF under different conditions. For example, one line might represent MTF values when the lens is at an aperture of f/4.0, while another might represent MTF values at f/8.0. A big hurdle with understanding how to read an MTF chart is learning what each line refers to.

Each line above has three different styles: thickness, color and type. Here's a breakdown of what each of these represents:

Line Thickness:	Bold → 10 LP/mm - small-scale contrast<br>Thin → 30 LP/mm - resolution or fine detail
Line Color:	Blue → Aperture at f/8.0<br>Black → Aperture wide open
Line Type:	Dashed → Meridional (concentric) line pairs<br>Solid → Sagittal (radial) line pairs

Since a given line can have any combination of thickness, color and type, the above MTF chart has a total of eight different types of lines. For example, a curve that is bold, blue and dashed would describe the MTF of meridional 10 LP/mm lines at an aperture of f/8.0.

Black Lines. These are most relevant when you are using your lens in low light, need to freeze rapid movement, or need a shallow depth of field. The MTF of black lines will almost always be a worst case scenario (unless you use unusually small apertures).

In the above example, black lines unfortunately aren't a fair apples to apples comparison, since a wide open aperture is different for each of the above lenses (f/2.8 on the zoom vs f/1.4 on the prime). This is the main reason why the black lines appear so much worse for the prime lens. However, given that the prime lens has such a handicap, it does quite admirably — especially at 10 LP/mm in the center, and at 30 LP/mm toward the edges of the image. It's therefore highly likely that the prime lens will outperform the zoom lens when they're both at f/2.8, but we cannot say for sure based only on the above charts.

Blue Lines. These are most relevant for landscape photography, or other situations where you need to maximize depth of field and sharpness. They are also more useful for comparisons because blue lines are always to be at the same aperture: f/8.0.

In the above example, the prime lens has a better MTF at all positions, for both high and low frequency details (30 and 10 LP/mm). The advantage of the prime lens is even more pronounced towards the outer regions of the camera's image.

Bold vs. Thin Lines. Bold lines describe the amount of "pop" or small-scale contrast, whereas thin lines describe finer details or resolution. Bold lines are often a priority since high values can mean that your images will have a more three dimensional look, similar to what happens when performing local contrast enhancement.

In the above example, both lenses have similar contrast at f/8.0, although the prime lens is a little better here. The zoom lens barely loses any contrast when used wide open compared to at f/8.0. On the other hand, the prime lens loses quite a bit of contrast when going from f/8.0 to f/1.4, but this is probably because f/1.4-f/8.0 is a much bigger change than f/2.8-f/8.0.

ASTIGMATISM: SAGITTAL vs. MERIDIONAL LINES

Dashed vs. Solid Lines. At this point you're probably wondering: why show the MTF for both sagittal ("S") and meridional ("M") line pairs? Wouldn't these be the same? Yes, at the image's direct center they're always identical. However, things become more interesting progressively farther from the center. Whenever the dashed and solid lines begin to diverge, this means that the amount of blur is not equal in all directions. This quality-reducing artifact is called an "**astigmatism**," as illustrated below:

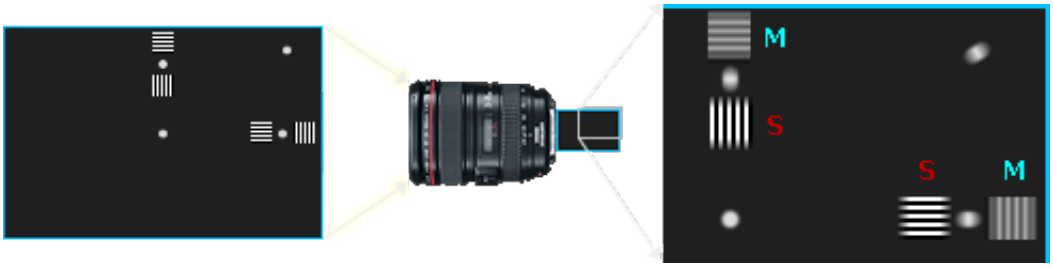

Select Aberration Type:

Astigmatism: MTF in S > M
Astigmatism: MTF in M > S
No Astigmatism: MTF in M=S

Move your mouse over the labels on the image to the right to see the effect of astigmatism.
S = sagittal lines, M = meridional lines
Note: Technically, the S above will have a slightly better MTF because it is closer to the center of the image; however, for the purposes of this example we're assuming that M & S are at similar positions.

When the MTF in S is greater than in M, objects are blurred primarily along lines radiating out from the center of the image. In the above example, this causes the white dots to appear to streak outward from the center of the image, almost as if they had motion blur. Similarly, objects are blurred in the opposite (circular) direction when the MTF in M is greater than in S. Many of you reading this tutorial right now might even be using eye glasses that correct for an astigmatism...

Technical Note: With wide angle lenses, M lines are much more likely to have a lower MTF than S lines, partly because these try to preserve a rectilinear image projection. Therefore, as the angle of view becomes wider, subjects near the periphery become progressively more stretched/distorted in directions leading away from the center of the image. A wide angle lens with significant barrel distortion can therefore achieve a better MTF since objects at the periphery are stretched much less than they would be otherwise. However, this is usually an unacceptable trade-off with architectural photography.

In the MTF charts for the Canon zoom versus prime lens from before, both lenses begin to exhibit pronounced astigmatism at the very edges of the image. However, with the prime lens, something interesting happens: the type of astigmatism reverses when comparing the lens at f/1.4 versus at f/8.0. At f/8.0, the lens primarily blurs in the radial direction, which is a common occurrence. However, at f/1.4 the prime lens primarily blurs in a circular direction, which is much less common.

What does astigmatism mean for your photos? Probably the biggest implication, other than the unique appearance, is that standard sharpening tools may not work as intended. These tools assume that blur is equal in all directions, so you might end up over-sharpening some edges, while leaving other edges still looking blurry. Astigmatism can also be problematic with photos containing stars or other point light sources, since this will make the asymmetric blur more apparent.

MTF & APERTURE: FINDING THE "SWEET SPOT" OF A LENS

The MTF of a lens typically increases for successively narrower apertures, then reaches a maximum for intermediate apertures, and finally declines again for very narrow apertures. The figure below shows the MTF-50 for various apertures on a high-quality lens:

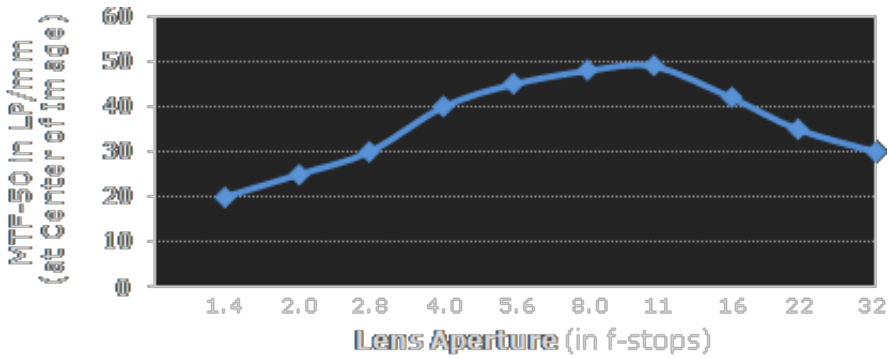

The aperture corresponding to the maximum MTF is the so-called "sweet spot" of a lens, since images will generally have the best sharpness and contrast at this setting. On a full frame or cropped sensor camera, this sweet spot is usually somewhere between f/8.0 and f/16, depending on the lens. The location of this sweet spot is also independent of the number of megapixels in your camera.

Technical Notes:

- At large apertures, resolution and contrast are typically limited by light aberrations.
  An aberration is when imperfect lens design causes a point light source in the image not to converge onto a point on your camera's sensor.
- At small apertures, resolution and contrast are typically limited by diffraction.
  Unlike aberrations, diffraction is a fundamental physical limit caused by the scattering of light, and is not necessarily any fault of the lens design.
- High and low quality lenses are therefore very similar when used at small apertures
  (such as f/16-32 on a full frame or cropped sensor).
- Large apertures are where high quality lenses really stand out, because the materials and engineering of the lens are much more important. In fact, a perfect lens would not even have a "sweet spot"; the optimal aperture would just be wide open.

However, one should not conclude that the optimal aperture setting is completely independent of what is being photographed. The sweet spot at the center of the image may not correspond with where the edges and corners of the image look their best; this often requires going to an even narrower aperture. Furthermore, this all assumes that your subject is in perfect focus; objects outside the depth of field will likely still improve in sharpness even when your f-stop is larger than the so-called sweet spot.

COMPARING DIFFERENT CAMERAS & LENS BRANDS

A big problem with the MTF concept is that it's not standardized. Comparing different MTF charts can therefore be quite difficult, and in some cases even impossible. For example, MTF charts by Canon and Nikon cannot be directly compared, because the Canon uses theoretical calculations while Nikon uses measurements.

However, even if one performed their own MTF tests, they'd still run into problems. **A typical self-run MTF chart actually depicts the net total MTF of your camera's optical system** — and not the MTF of the lens alone. This net MTF represents the combined result from the lens, camera sensor and RAW conversion, in addition to any sharpening or other post-processing. MTF measurements will therefore vary depending on which camera is used for the measurement, or the type of software used in the RAW conversion. It's therefore only practical to compare MTF charts that were measured using identical methodologies.

Cropped vs. Full Frame Sensors. One needs to be extra careful when comparing MTF charts amongst

cameras with different sensor sizes. For example, an MTF curve at 30 LP/mm on a full frame camera is not equivalent to a different 30 LP/mm MTF curve on a 1.6X cropped sensor. The cropped sensor would instead need to show a curve at 48 LP/mm for a fair comparison, because the cropped sensor gets enlarged more when being made into the same size print.

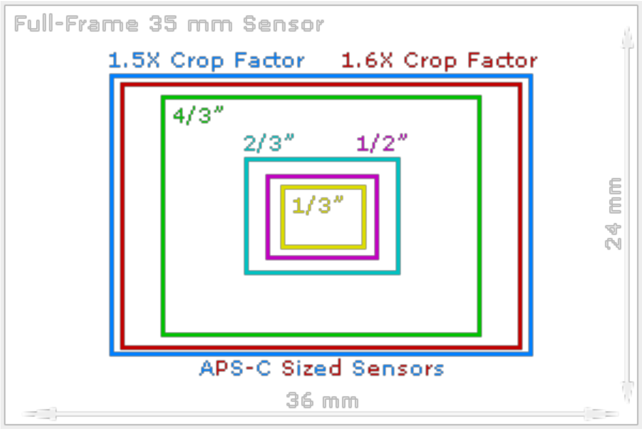

The diversity of sensor sizes is why some have started listing the line frequency in terms of the picture or image height (LP/PH or LP/IH), as opposed to using an absolute unit like a millimeter. A line frequency of 1000 LP/PH, for example, has the same appearance at a given print size — regardless of the size of the camera's sensor. One would suspect that part of the reason manufacturers keep showing MTF charts at 10 and 30 LP/mm for DX, EF-S and other cropped sensor lenses is because this makes their MTF charts look better.

MTF CHART LIMITATIONS

While MTF charts are an extremely powerful tool for describing the quality of a lens, they still have many limitations. In fact, an MTF chart says nothing about:

- Color quality and chromatic aberrations
- Image distortion
- Vignetting (light fall-off toward the edges of an image)
- Susceptibility to camera lens flare

Furthermore, other factors such as the condition of your equipment and your camera technique can often have much more of an impact on the quality of your photos than small differences in the MTF. Some of these quality-reducing factors might include:

- Focusing accuracy
- Camera shake
- Dust on your camera's digital sensor (see tutorial on camera sensor cleaning)
- Micro abrasions, moisture, fingerprints or other coatings on your lens

Most importantly, even though MTF charts are amazingly sophisticated and descriptive tools — with lots of good science to back them up — ultimately nothing beats simply visually inspecting an image on-screen or in a print. After all, pictures are made to look at, so that's all that really matters at the end of the day. It can often be quite difficult to discern whether an image will look better on another lens based on an MTF, because there's usually many competing factors: contrast, resolution, astigmatism, aperture, distortion, etc. A lens is rarely superior in all of these aspects at the same time. If you cannot tell the different between shots with different lenses used in similar situations, then any MTF discrepancies

probably don't matter.

Finally, even if one lens's MTF is indeed worse than another's, sharpening and local contrast enhancement can often make this disadvantage imperceptible in a print — as long as the original quality difference isn't too great.

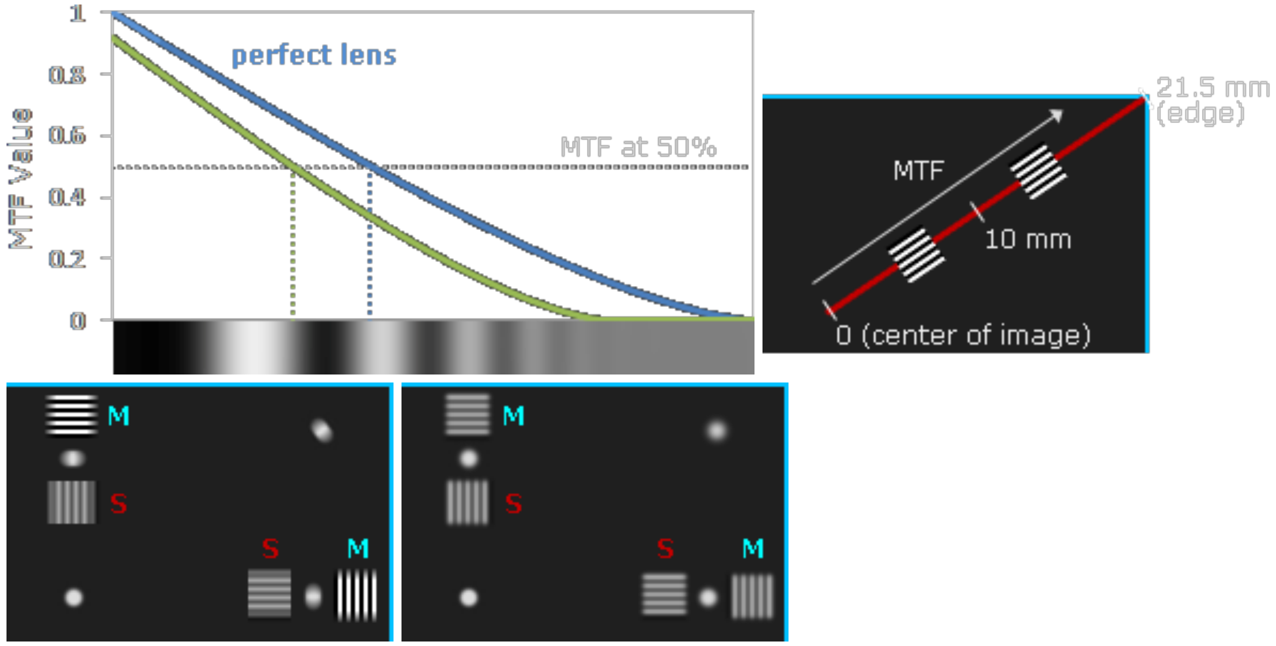

Want to learn more? Discuss this and other articles in our digital photography forums.

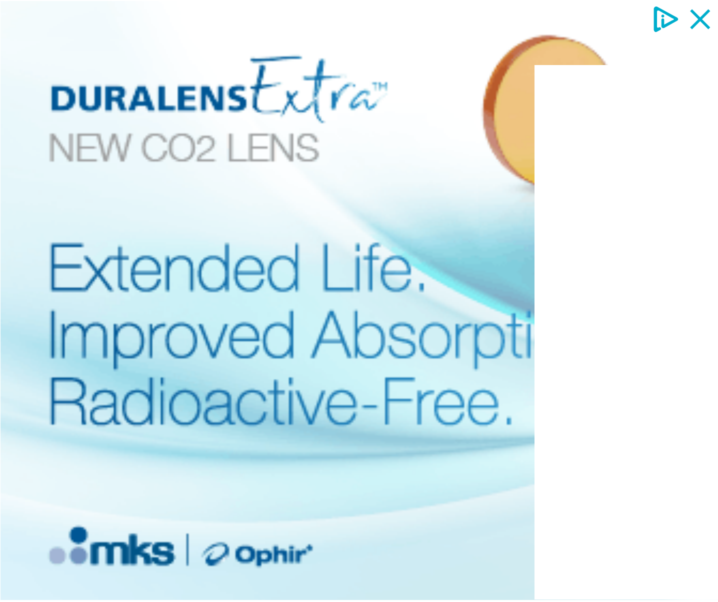

- Back to Photography Tutorials -

Follow us via

- Email
- Facebook
- Twitter

BROWSE CATEGORIES

- Concepts & Terminology
- Using Camera Equipment
- Editing & Post-Processing
- Color Management & Printing
- Photo Techniques & Styles

GET OUR NEW BOOK!

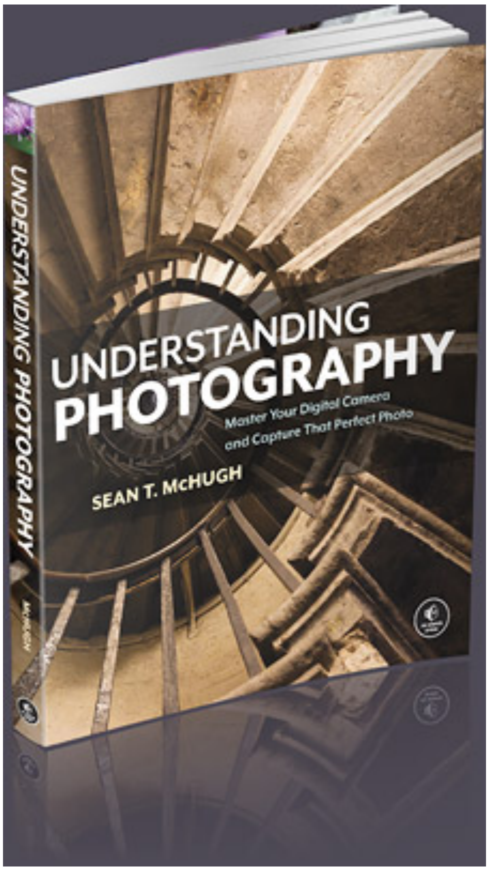

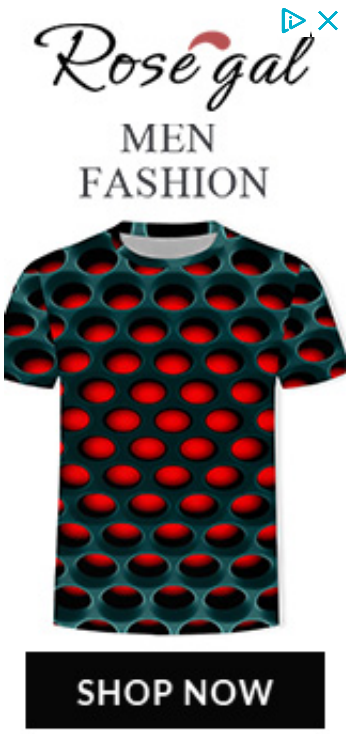

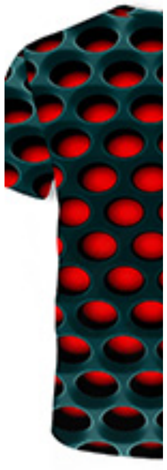

11
Gefäl

Copyright © 2005-2019 Cambridge in Colour|Gallery|About|Contact Us

- Email
- Facebook
- Twitter
