# Supplementary material for: Experimental setup and image processing method for automatic enumeration of bacterial colonies on agar plates
Source: PLoS One. 2020 Jun 24;15(6):e0232869. doi: 10.1371/journal.pone.0232869 (PMC7313745; doi:10.1371/journal.pone.0232869)
Supplement: S2 File — (PDF) [file pone.0232869.s003.pdf]

ij.plugin.filter

Class MaximumFinder

java.lang.Object
    ij.plugin.filter.MaximumFinder

All Implemented Interfaces:

`DialogListener, ExtendedPlugInFilter, PlugInFilter`

```
public class MaximumFinder
extends java.lang.Object
implements ExtendedPlugInFilter, DialogListener
```

This ImageJ plug-in filter finds the maxima (or minima) of an image. It can create a mask where the local maxima of the current image are marked (255; unmarked pixels 0). The plug-in can also create watershed-segmented particles: Assume a landscape of inverted heights, i.e., maxima of the image are now water sinks. For each point in the image, the sink that the water goes to determines which particle it belongs to. When finding maxima (not minima), pixels with a level below the lower threshold can be left unprocessed. Except for segmentation, this plugin works with area ROIs, including non-rectangular ROIs, which define the area where maxima are reported. Since this plug-in creates a separate output image it processes only single images or slices, no stacks. Notes: - When using one instance of MaximumFinder for more than one image in parallel threads, all must images have the same width and height. version 09-Nov-2006 Michael Schmid version 21-Nov-2006 Wayne Rasband. Adds "Display Point Selection" option and "Count" output type. version 28-May-2007 Michael Schmid. Preview added, bugfix: minima of calibrated images, uses Arrays.sort version 07-Aug-2007 Fixed a bug that could delete particles when doing watershed segmentation of an EDM. version 21-Apr-2007 Adapted for float instead of 16-bit EDM; correct progress bar on multiple calls version 05-May-2009 Works for images>32768 pixels in width or height version 01-Nov-2009 Bugfix: extra lines in segmented output eliminated; watershed is also faster now Maximum points encoded in long array for sorting instead of separete objects that need gc New output type 'List' version 22-May-2011 Bugfix: Maximum search in EDM and float images with large dynamic range could omit maxima version 13-Sep-2013 added the findMaxima() and findMinima() functions for arrays (Norbert Vischer) version 20-Mar-2014 Watershed segmentation of EDM with tolerance>=1.0 does not kill fine particles version 11-Mar-2019 adds "strict" option, "noise tolerance" renamed to "prominence"

Field Summary

Fields

Modifier and Type	Field and Description

static int	**COUNT**<br>Do not create an image, just count maxima and add count to Results table
static int	**IN_TOLERANCE**<br>Output type all points around the maximum within the tolerance
static int	**LIST**<br>Do not create an image, just list x, y of maxima in the Results table
static int	**POINT_SELECTION**<br>Do not create image, only mark points
static int	**SEGMENTED**<br>Output type watershed-segmented image
static int	**SINGLE_POINTS**<br>Output type single points

Fields inherited from interface ij.plugin.filter.ExtendedPlugInFilter

KEEP_PREVIEW

Fields inherited from interface ij.plugin.filter.PlugInFilter

CONVERT_TO_FLOAT, DOES_16, DOES_32, DOES_8C, DOES_8G, DOES_ALL, DOES_RGB, DOES_STACKS, DONE, FINAL_PROCESSING, KEEP_THRESHOLD, NO_CHANGES, NO_IMAGE_REQUIRED, NO_UNDO, NO_UNDO_RESET, PARALLELIZE_IMAGES, PARALLELIZE_STACKS, ROI_REQUIRED, SNAPSHOT, STACK_REQUIRED, SUPPORTS_MASKING

Constructor Summary

Constructors

Constructor and Description
**MaximumFinder**()

Method Summary

All Methods | **Static Methods** | **Instance Methods** | **Concrete Methods**

Modifier and Type	Method and Description
boolean	**dialogItemChanged**(**GenericDialog** gd, java.awt.AWTEvent e)<br>Read the parameters (during preview or after showing the dialog)

static int[]	**findMaxima**(double[] xx, double tolerance, boolean excludeOnEdges)
static int[]	**findMaxima**(double[] xx, double tolerance, int edgeMode)<br>Calculates peak positions of 1D array N.Vischer, 06-mar-2017
**ByteProcessor**	**findMaxima**(**ImageProcessor** ip, double tolerance, boolean strict, double threshold, int outputType, boolean excludeOnEdges, boolean isEDM)<br>Here the processing is done: Find the maxima of an image (does not find minima).
**ByteProcessor**	**findMaxima**(**ImageProcessor** ip, double tolerance, double threshold, int outputType, boolean excludeOnEdges, boolean isEDM)<br>Finds the maxima of an image (does not find minima).
**ByteProcessor**	**findMaxima**(**ImageProcessor** ip, double tolerance, int outputType, boolean excludeOnEdges)<br>Find the maxima of an image.
static int[]	**findMinima**(double[] xx, double tolerance, boolean excludeEdges)<br>Returns minimum positions of array xx, sorted with decreasing strength
static int[]	**findMinima**(double[] xx, double tolerance, int edgeMode)
java.awt.Polygon	**getMaxima**(**ImageProcessor** ip, double tolerance, boolean excludeOnEdges)<br>Finds the image maxima and returns them as a Polygon, where poly.npoints is the number of maxima.
java.awt.Polygon	**getMaxima**(**ImageProcessor** ip, double tolerance, boolean strict, boolean excludeOnEdges)<br>Finds the image maxima and returns them as a Polygon, where poly.npoints is the number of maxima.
void	**run**(**ImageProcessor** ip)<br>The plugin is inferred from ImageJ by this method
void	**setNPasses**(int nPasses)<br>Set his to the number of images to process (for the watershed progress bar only).
int	**setup**(java.lang.String arg, **ImagePlus** imp)<br>Method to return types supported
int	**showDialog**(**ImagePlus** imp, java.lang.String command, **PlugInFilterRunner** pfr)<br>This method is called after `setup(arg, imp)` unless the `DONE` flag has been set.

Methods inherited from class java.lang.Object

`clone, equals, finalize, getClass, hashCode, notify, notifyAll, toString, wait, wait, wait`

Field Detail

SINGLE_POINTS

`public static final int SINGLE_POINTS`

Output type single points

See Also:

Constant Field Values

IN_TOLERANCE

`public static final int IN_TOLERANCE`

Output type all points around the maximum within the tolerance

See Also:

Constant Field Values

SEGMENTED

`public static final int SEGMENTED`

Output type watershed-segmented image

See Also:

Constant Field Values

POINT_SELECTION

`public static final int POINT_SELECTION`

Do not create image, only mark points

See Also:

Constant Field Values

LIST

`public static final int LIST`

Do not create an image, just list x, y of maxima in the Results table

See Also:

`Constant Field Values`

COUNT

```
public static final int COUNT
```

Do not create an image, just count maxima and add count to Results table

See Also:

`Constant Field Values`

Constructor Detail

MaximumFinder

```
public MaximumFinder()
```

Method Detail

setup

```
public int setup(java.lang.String arg,
                 ImagePlus imp)
```

Method to return types supported

Specified by:

`setup` in interface `PlugInFilter`

Parameters:

`arg` - Not used by this plugin-filter

`imp` - The image to be filtered

Returns:

Code describing supported formats etc. (see ij.plugin.filter.PlugInFilter & ExtendedPlugInFilter)

showDialog

```
public int showDialog(ImagePlus imp,
                      java.lang.String command,
```

```
                      PlugInFilterRunner pfr)
```

Description copied from interface: `ExtendedPlugInFilter`

This method is called after `setup(arg, imp)` unless the `DONE` flag has been set.

Specified by:

`showDialog` in interface `ExtendedPlugInFilter`

Parameters:

`imp` - The active image already passed in the setup(arg, imp) call. It will be null, however, if the NO_IMAGE_REQUIRED flag has been set.

`command` - The command that has led to the invocation of the plugin-filter. Useful as a title for the dialog.

`pfr` - The PlugInFilterRunner calling this plugin-filter. It can be passed to a GenericDialog by addPreviewCheckbox to enable preview by calling the run(ip) method of this plugin-filter. pfr can be also used later for calling back the PlugInFilterRunner, e.g., to obtain the slice number currently processed by run(ip).

Returns:

The method should return a combination (bitwise OR) of the flags specified in interfaces PlugInFilter and ExtendedPlugInFilter.

dialogItemChanged

```
public boolean dialogItemChanged(GenericDialog gd,
                                 java.awt.AWTEvent e)
```

Read the parameters (during preview or after showing the dialog)

Specified by:

`dialogItemChanged` in interface `DialogListener`

Parameters:

`gd` - A reference to the GenericDialog.

`e` - The event that has been generated by the user action in the dialog. Note that e is null if the dialogItemChanged method is called after the user has pressed the OK button or if the GenericDialog has read its parameters from a macro.

Returns:

Should be true if the dialog input is valid. False disables the OK button and preview (if any).

setNPasses

```
public void setNPasses(int nPasses)
```

Set his to the number of images to process (for the watershed progress bar only). Don't call or set nPasses to zero if no progress bar is desired.

Specified by:

`setNPasses` in interface `ExtendedPlugInFilter`

run

```
public void run(ImageProcessor ip)
```

The plugin is inferred from ImageJ by this method

Specified by:

`run` in interface `PlugInFilter`

Parameters:

`ip` - The image where maxima (or minima) should be found

getMaxima

```
public java.awt.Polygon getMaxima(ImageProcessor ip,
                                  double tolerance,
                                  boolean excludeOnEdges)
```

Finds the image maxima and returns them as a Polygon, where poly.npoints is the number of maxima. There is an example at http://imagej.nih.gov/ij/macros/js/FindMaxima.js.

Parameters:

`ip` - The input image

`tolerance` - Height tolerance: maxima are accepted only if protruding more than this value from the ridge to a higher maximum

`excludeOnEdges` - Whether to exclude edge maxima. Also determines whether strict mode is on, i.e., whether the global maximum is accepted even if all other pixel are less than 'tolerance' below this level (In 1.52m and before, 'strict' and 'excludeOnEdges' were the same).

Returns:

A Polygon containing the coordinates of the maxima, where poly.npoints is the number of maxima. Note that poly.xpoints.length may be greater than the number of maxima.

getMaxima

```
public java.awt.Polygon getMaxima(ImageProcessor ip,
                                  double tolerance,
                                  boolean strict,
                                  boolean excludeOnEdges)
```

Finds the image maxima and returns them as a Polygon, where poly.npoints is the number of

maxima.

Parameters:

ip - The input image

tolerance - Height tolerance: maxima are accepted only if protruding more than this value from the ridge to a higher maximum

strict - When off, the global maximum is accepted even if all other pixel are less than 'tolerance' below this level. With excludeOnEdges=true, 'strict' also means that the surounding of a maximum within 'tolerance' must not include an edge pixel (otherwise, it is enough that there is no edge pixel with the maximum value).

excludeOnEdges - Whether to exclude edge maxima. Also determines whether strict mode is on, i.e., whether the global maximum is accepted even if all other pixel are less than 'tolerance' below this level (In 1.52m and before, 'strict' and 'excludeOnEdges' were the same).

Returns:

A Polygon containing the coordinates of the maxima, where poly.npoints is the number of maxima. Note that poly.xpoints.length may be greater than the number of maxima.

findMaxima

```
public static int[] findMaxima(double[] xx,
                               double tolerance,
                               int edgeMode)
```

Calculates peak positions of 1D array N.Vischer, 06-mar-2017

Parameters:

xx - Array containing peaks.

tolerance - Depth of a qualified valley must exceed tolerance. Tolerance must be >= 0. Flat tops are marked at their centers.

edgeMode - 0=include, 1=exclude, 3=circular edgeMode = 0 (include edges) peak may be separated by one qualified valley and by a border. edgeMode = 1 (exclude edges) peak must be separated by two qualified valleys edgeMode = 2 (circular) array is regarded to be circular

Returns:

Positions of peaks, sorted with decreasing amplitude

findMaxima

```
public static int[] findMaxima(double[] xx,
                               double tolerance,
                               boolean excludeOnEdges)
```

findMinima

```
public static int[] findMinima(double[] xx,
                               double tolerance,
                               boolean excludeEdges)
```

Returns minimum positions of array xx, sorted with decreasing strength

findMinima

```
public static int[] findMinima(double[] xx,
                               double tolerance,
                               int edgeMode)
```

findMaxima

```
public ByteProcessor findMaxima(ImageProcessor ip,
                                double tolerance,
                                int outputType,
                                boolean excludeOnEdges)
```

Find the maxima of an image.

Parameters:

ip - The input image

tolerance - Height tolerance: maxima are accepted only if protruding more than this value from the ridge to a higher maximum

outputType - What to mark in output image: SINGLE_POINTS, IN_TOLERANCE or SEGMENTED. No output image is created for output types POINT_SELECTION, LIST and COUNT.

excludeOnEdges - Whether to exclude edge maxima. Also determines whether strict mode is on, i.e., whether the global maximum is accepted even if all other pixel are less than 'tolerance' below this level (In 1.52m and before, 'strict' and 'excludeOnEdges' were the same).

Returns:

A new byteProcessor with a normal (uninverted) LUT where the marked points are set to 255 (Background 0). Pixels outside of the roi of the input ip are not set. Returns null if outputType does not require

an output or if cancelled by escape

findMaxima

```
public ByteProcessor findMaxima(ImageProcessor ip,
                                double tolerance,
                                double threshold,
                                int outputType,
                                boolean excludeOnEdges,
                                boolean isEDM)
```

Finds the maxima of an image (does not find minima). LIMITATIONS: With outputType=SEGMENTED (watershed segmentation), some segmentation lines may be improperly placed if local maxima are suppressed by the tolerance.

Parameters:

ip - The input image

tolerance - Height tolerance: maxima are accepted only if protruding more than this value from the ridge to a higher maximum

threshold - minimum height of a maximum (uncalibrated); for no minimum height set it to ImageProcessor.NO_THRESHOLD

outputType - What to mark in output image: SINGLE_POINTS, IN_TOLERANCE or SEGMENTED. No output image is created for output types POINT_SELECTION, LIST and COUNT.

excludeOnEdges - Whether to exclude edge maxima. Also determines whether strict mode is on, i.e., whether the global maximum is accepted even if all other pixel are less than 'tolerance' below this level (In 1.52m and before, 'strict' and 'excludeOnEdges' were the same).

isEDM - Whether the image is a float Euclidian Distance Map.

Returns:

A new byteProcessor with a normal (uninverted) LUT where the marked points are set to 255 (Background 0). Pixels outside of the roi of the input ip are not set. Returns null if outputType does not require an output or if cancelled by escape

findMaxima

```
public ByteProcessor findMaxima(ImageProcessor ip,
                                double tolerance,
                                boolean strict,
                                double threshold,
                                int outputType,
                                boolean excludeOnEdges,
                                boolean isEDM)
```

Here the processing is done: Find the maxima of an image (does not find minima). LIMITATIONS: With outputType=SEGMENTED (watershed segmentation), some segmentation lines may be improperly placed if local maxima are suppressed by the tolerance.

Parameters:

`ip` - The input image

`tolerance` - Height tolerance: maxima are accepted only if protruding more than this value from the ridge to a higher maximum

`strict` - When off, the global maximum is accepted even if all other pixel are less than 'tolerance' below this level. With excludeOnEdges=true, 'strict' also means that the surounding of a maximum within 'tolerance' must not include an edge pixel (otherwise, it is enough that there is no edge pixel with the maximum value).

`threshold` - Minimum height of a maximum (uncalibrated); for no minimum height set it to ImageProcessor.NO_THRESHOLD

`outputType` - What to mark in output image: SINGLE_POINTS, IN_TOLERANCE or SEGMENTED. No output image is created for output types POINT_SELECTION, LIST and COUNT.

`excludeOnEdges` - Whether to exclude edge maxima

`isEDM` - Whether the image is a float Euclidian Distance Map.

Returns:

A new byteProcessor with a normal (uninverted) LUT where the marked points are set to 255 (Background 0). Pixels outside of the roi of the input ip are not set. Returns null if outputType does not require an output or if cancelled by escape
