# Supplementary material for: Experimental setup and image processing method for automatic enumeration of bacterial colonies on agar plates
Source: PLoS One. 2020 Jun 24;15(6):e0232869. doi: 10.1371/journal.pone.0232869 (PMC7313745; doi:10.1371/journal.pone.0232869)
Supplement: S3 File — (PDF) [file pone.0232869.s004.pdf]

▶ Community Partners

New maxima finder menu in FIJI

fiji

---

gayaJ **May 9**

Hello!

So the Maxima finder menu seems to have been updated in FIJI which I find better than the previous one, thank you! But I would love to know what the option “Strict” does. I did a quick test with blobs image and checking and unchecking this box did not affect the number of maxima detected. Does anyone have any idea?

Thanks!

---

bogovicj Community Forum Team member **May 9**

Hi **@gayaJ** ,

From **here** :

A little tough for me to interpret, especially because it interacts with “exclude edges” but:

“Strict”:

> When off, the global maximum is accepted even if all other pixel are less than ‘tolerance’ below this level. With excludeOnEdges=true, ‘strict’ also means that the surounding of a maximum within ‘tolerance’ must not include an edge pixel (otherwise, it is enough that there is no edge pixel with the maximum value).

“Exclude edges”:

> Whether to exclude edge maxima. Also determines whether strict mode is on, i.e., whether the global maximum is accepted even if all other pixel are less than ‘tolerance’ below this level (In 1.52m and before, ‘strict’ and ‘excludeOnEdges’ were the same).

John

schmiedc May 9

I tested it with the blobs.gif sample.
Light background was turned on.
When “prominence” was set to 100.

Strict turned off
exclude edge maxima turned off
61 Maxima

Strict turned on
exclude edge maxima turned off
61 Maxima

Strict turned off
exclude edge maxima turned on
50 Maxima

Strict turned on
exclude edge maxima turned on
43 Maxima

Strict seems thus to be a sub rule of exclude edge maxima?

---

schmiedc May 9

Why is the parameter in the code actually called tolerance and in the GUI there is the word prominence?

Compared to its ease of use the Maxima Finder must be one of the most convolved and weirdly described plugins of this software…
I don’t know how a Biologist should understand any of this…

---

schmid May 9

Hi almost-namesake,

concerning the name of the parameter: Originally it was called “tolerance”, but it was renamed to “prominence” when the behavior was modified in 1.52m. This was necessary for backwards compatibility, so the old behavior old macros with the keyword “tolerance” still get the same behavior as previously, when “strict” and “exclude on edges” were coupled. The new keyword “prominence” is in analogy to topographic prominence in geography:
https://en.wikipedia.org/wiki/Topographic_prominence
Replacing “tolerance” with “prominence” in the code at the same time would have made it impossible to

compare the new and old code and see what has changed, because there would be too many changes.

Here is a bit more explanation for how “strict” and “exclude edge maxima” determine what you get:

- If “exclude edge maxima” is off, “strict=on” means that we get no maximum (not one) if there is no pixel below the ‘max - prominence’ limit. So, with prominence=8000 meters, Mount Everest (8850 m) would be a maximum in a map of Asia including the sea shore, but not in a map of 1 km around its summit. With “strict=off”, the peak will be reported as a maximum for both maps.
- If “exclude edge maxima” is on, “strict” also means that from the peak to any edge there must be a valley reaching down to the ‘maximum minus prominence’ level. With prominence=8000 m, Mt Everest would not qualify as a maximum in a map of India and Southern China, because there will be a continuous path above 8850 m - 8000 m = 850 m from the peak to the edge of the map at the north (through the highlands of Southern China).
- With “exclude edge maxima=on”, “strict=off”, and prominence=8000 m, Mt. Everest would be the only peak in that map, unless its peak is **exactly** at the border. K2 (8611 m) would be still suppressed if Mt. Everest is in the field, because its prominence is only 4020 m, i.e. one can go 4020 m down from K2 and then ascend to Mt. Everest, which is higher.

Does this excursion into geography make it more clear?

–Michael

🔗 **Counting neurons**

🔗 **BAR findPeaks plugin not detecting the same peaks on two computer**

schmiedc **May 10**

Ahhhh awesome! Yes that cleared things up tremendously!

So if I get this correctly if “prominence=8000”, “exclude edge maxima=off” and “strict=off” a maximum would be accepted from >8000 even if the minimum value in the image is 1000. So the peak would be calculated from 0 to the peak.

If “prominence=8000”, “exclude edge maxima=off” and “strict=on” the calculation would be from the base of the mountain to the peak only.

I see now that the strict parameter is an important and very useful parameter.

Thanks a lot!

schmid **May 10**

Hi,

maybe one more thing that might be helpful:

With “strict=off”, one can use “Find Maxima” for finding the highest peak in an image. Just set the prominence to a value that is higher than the dynamic range of the image (even a value of 1e20 does not hurt). No maximum will be reported with these settings if the highest peak does not fulfill some other criterion (e.g. “exclude edge maxima=on” and the highest pixel is at the edge, or the maximum consists of several pixels of equal value and one of them is at the edge, or the maximum is not in the current selection). Also, no maximum is reported if all pixels of the image are equal.

–Michael

---

schmiedc **May 10**

It would be great if this could be documented in the wiki.
I am more than willing to create this also, under the condition that you would correct it of course.

Or is there a plan to update the ImageJ User Guide … or do a documentation in an other way?

Thank you!

---

mverp **May 13**

Hi,

Thanks for the clear explanation of prominence and the dialog parameters.

One question though I got from experimenting with these.
When I use the standard sample ‘blobs’ image and use the Find Maxima function I got a strange result with the following settings:

- If I use a prominence of >200 and a light background I get 4 maxima , so far so good.
- When I now switch on Exclude edge maxima (but not Strict), I lose two of the maxima. However one of these (167,143) is very certainly not on the edge. As a matter of fact, it is the one furthest from the edge of the four maxima originally found.

Is there something I am missing about the ‘Exclude on edge’ setting?
