# Supplementary material for: Experimental setup and image processing method for automatic enumeration of bacterial colonies on agar plates
Source: PLoS One. 2020 Jun 24;15(6):e0232869. doi: 10.1371/journal.pone.0232869 (PMC7313745; doi:10.1371/journal.pone.0232869)
Supplement: S4 File — (PDF) [file pone.0232869.s005.pdf]

Xenon-Ruby 2.3/16

Schneider KREUZNACH

The Xenon-Ruby is a lightweight compact c-mount lens for 1/1.8" sensor up to 10 megapixel resolution with 16 mm focal length. Robust, with lockable focus and iris setting this lens is vibration insensitivity guarantees a stable image performance that makes Xenon-Ruby high usable under industrial conditions.

The possibility to use this wide angle lens from near 70 mm working distance to infinity offers many opportunities to be implemented in barcode scanning systems in logistic center or aside a conveyer band of sorting systems. Equipped with 400 nm -1000 nm broadband AR- coating this lens functions for security and surveillance duty. Lenses with 10 mm, 25 mm and 35 mm focal length complete the Xenon-Ruby series.

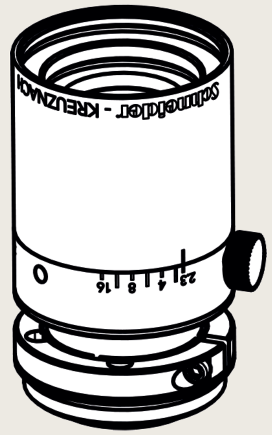

Xenon-Ruby 2.3/16

Key features

- For 1/1.8" sensors up to 10 megapixel
- 55 g lightweight mode of construction
- 400 nm to 1000 nm broadband AR coating
- Vibration insensitivity for stable imaging performance

Applications

- Bar code scanner
- Sorting system
- Security and surveillance

Name	Xenon-Ruby 2.3/16
Type	-0002
Focal Length [mm]	16
Magnification	-0.1
Image circle [mm]	9
Resolution [µm]	3.65
F/# range	2.3 ... 16
NA	0.22
Interface	C-Mount
Working distance [mm]	153
AoV [°]	31
Focus control	manual
Transmission [nm]	400 - 1000
Filter thread [mm]	M 25.5 x 0.5
Dimensions L x D [mm]	46.6 x 27.0

Name	Xenon-Ruby 2.3/16
Weight [g]	65
Storage temperature [°C]	-25 … +70
f'_{eff} [mm]	15.90
S_F [mm]	9.15
$S'_{F'}$ [mm]	17.05
HH' [mm]	15.92
β'_P	1.446
S_{EP} [mm]	20.15
$S'A_P$ [mm]	-5.93
Σd [mm]	39.81
ID	1074626

© Jos. Schneider Optische Werke GmbH I Version 1.0 10/2018 I Jos. Schneider Optische Werke GmbH is certified ISO 9001.

Xenon-Ruby 2.3/16

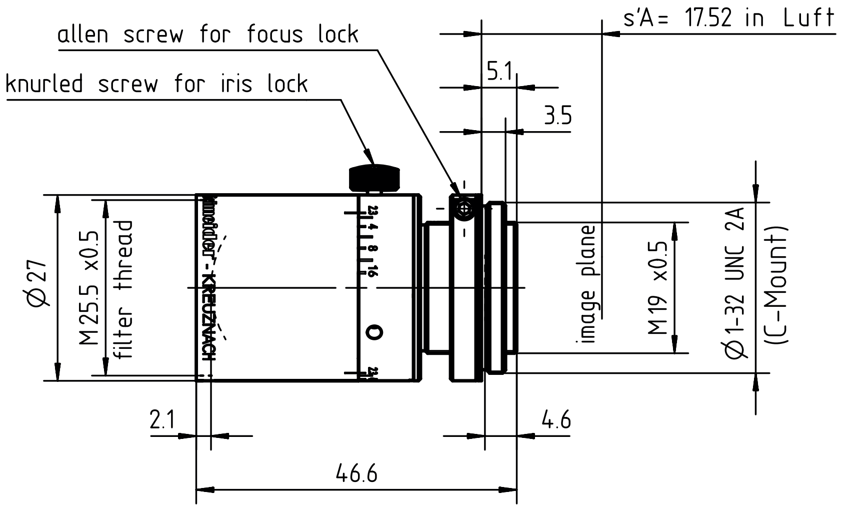

Distortion vs. Image Height

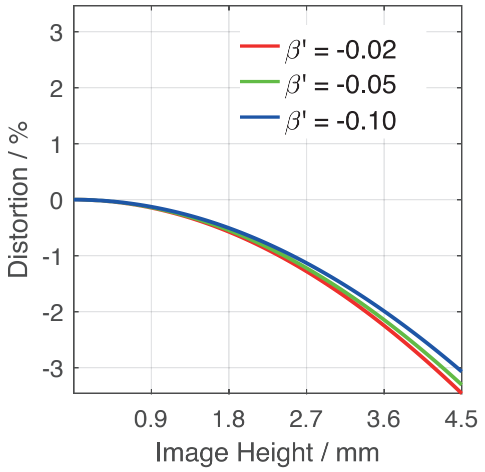

Transmittance vs. Wavelength

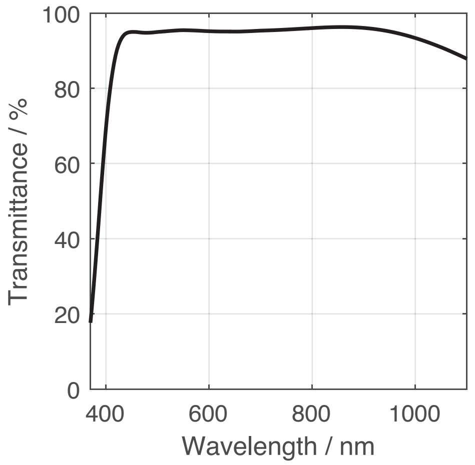

Relative Illumination vs. Image Height

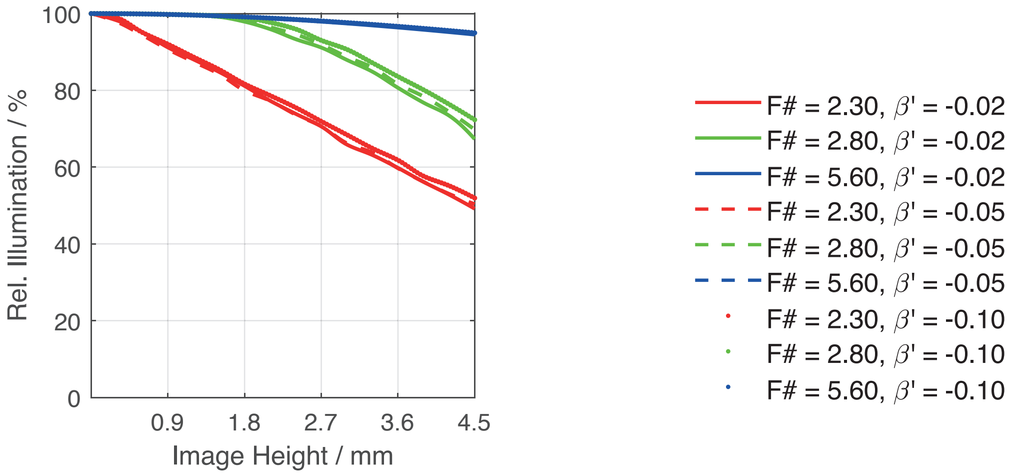

© Jos. Schneider Optische Werke GmbH | Version 1.0 10/2018 | Jos. Schneider Optische Werke GmbH is certified ISO 9001.

Xenon-Ruby 2.3/16

Schneider KREUZNACH

Spectrum Name	VIS					
Wavelenghts [nm]	425	475	525	575	625	675
Weights	8	16	23	22	19	13

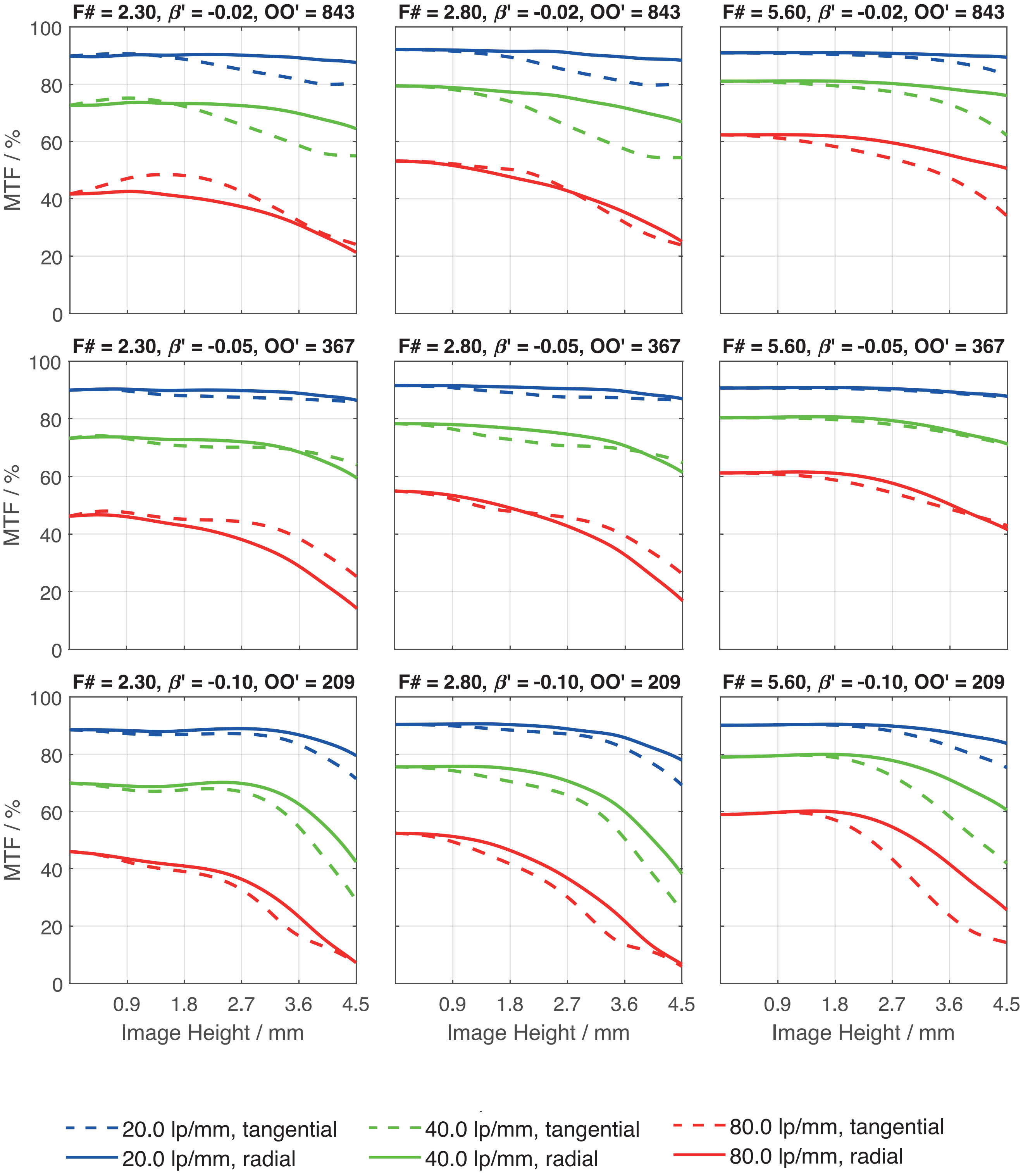

© Jos. Schneider Optische Werke GmbH | Version 1.0 10/2018 | Jos. Schneider Optische Werke GmbH is certified ISO 9001.

Accessories	Mount	Length	ID
Adapter	CS-Mount Adapter	5 mm	25081
	C-Mount / M42 x 1 Adapter	-	1075817
Ext. Tube	C-Mount	5 mm	39316
	C-Mount	8 mm	39315
	C-Mount	10 mm	39312

© Jos. Schneider Optische Werke GmbH | Version 1.0 10/2018 | Jos. Schneider Optische Werke GmbH is certified ISO 9001.

Get more information

Contact our local offices

Headquarter Germany

Jos. Schneider Optische Werke GmbH
Ringstraße 132
55543 Bad Kreuznach
☎ +49 671 601 205
✉ cs@schneiderkreuznach.com
www.schneiderkreuznach.com

Offices Worldwide

China
☎ +86 755 8832 1170
✉ shawn.liu@schneider-asiapacific.com

North America
☎ +1 800 645 7239 (East Coast)
☎ +1 800 228 1254 (West Coast)
✉ info@schneideroptics.com

Singapore
☎ +65 9488 0062
✉ thongv@schneiderkreuznach.com

© Jos. Schneider Optische Werke GmbH | Version 1.0 10/2018 | Jos. Schneider Optische Werke GmbH is certified ISO 9001.
